# Metallic (Al and Fe) Powder-Reinforced Styrene–Butadiene Rubber Composites for Triboelectric Energy Harvesting

**DOI:** 10.3390/polym18151801

**Published:** 2026-07-23

**Authors:** Md Najib Alam, Vishnu Shankar Dhandapani, Sang-Shin Park

**Affiliations:** School of Mechanical Engineering, Yeungnam University, 280 Daehak-ro, Gyeongsan 38541, Republic of Korea; mdnajib.alam3@gmail.com (M.N.A.); visshank@gmail.com (V.S.D.)

**Keywords:** metallic fillers, rubber composites, physical properties, triboelectric nanogenerator, metal–rubber interactions

## Abstract

This study explores the energy-harvesting performance of aluminum (Al)- and iron (Fe)-filled styrene–butadiene rubber (SBR) composites, with a focus on their mechanical durability and triboelectric properties. Comprehensive mechanical characterization—including tensile strength, elongation at break, fracture toughness, and elasticity—reveals that Fe-filled composites exhibit significantly enhanced reinforcement compared to Al-filled systems at equivalent filler loadings. Raman spectroscopy indicates that Fe atoms can coordinate with the benzene rings of SBR chains through stronger physicochemical bonding, a feature less present in Al-based composites. In addition to improved mechanical properties, Fe-filled composites demonstrate higher electrical conductivity and superior triboelectric energy-harvesting performance. Notably, the composite containing 15 vol% Fe under 1% cyclic compressive strain achieves a peak current density of 127.05 µA/m^2^, a total generated charge of 5.01 nC, and a peak power density of 48.22 µW/m^2^. These values represent substantial enhancements of 246%, 236%, and 2398%, respectively, compared to Al-filled counterparts. Cyclic energy-harvesting tests confirm stable performance with negligible degradation in output or mechanical integrity over repeated cycles. Rubber composite shows good humidity resistance in current and voltage outputs. Furthermore, a layer-by-layer triboelectric nanogenerator (TENG) based on the Fe-filled composite produces output signals of approximately ±1.0 µA and ±5 V under biomechanical hand patting. The superior performance of Fe-based composites is attributed to stronger filler–rubber interactions, likely facilitated by electrostatic interactions, which enhances interfacial charge transfer during mechanical deformation. Overall, Fe-filled SBR composites demonstrate strong potential for cost-effective, environmentally friendly, and durable self-powered energy-harvesting applications.

## 1. Introduction

Recently, various types of nanogenerators have attracted significant research interest [1,2]. Among them, triboelectric nanogenerators (TENGs) are particularly appealing due to their low material cost and the ubiquitous availability of mechanical energy in the environment. However, their energy conversion efficiency is generally considered lower than that of conventional power generators [3]. This reduced efficiency primarily arises from the unintentional conversion of input mechanical energy into secondary forms such as heat, light, and sound during triboelectrification, resulting in substantial energy losses [3]. To mitigate these losses, several strategies have been proposed, including the incorporation of organic–inorganic hybrid material systems, the integration of device architectures capable of harvesting diverse mechanical energy sources, and the development of user-friendly designs for industrial applications [3].

Fundamentally, triboelectrification generates surface charges that subsequently flow through an external conductive pathway. In the absence of such a pathway, accumulated charges tend to dissipate and transform into secondary energy forms, including heat, light, and sound. Therefore, to enhance TENG efficiency, a large electron affinity difference at the contact interface is essential for generating higher charge densities, and the constituent materials must possess sufficient electrical conductivity to enable efficient charge transport. Notably, thermal energy—particularly elevated temperatures—can adversely affect TENG performance [4].

Polymers play a crucial role in triboelectric nanogenerators due to their high dielectric constants, structural versatility, mechanical flexibility, and low cost, all of which contribute to improved energy conversion efficiency [5,6,7,8,9,10]. Rubber, a distinct class of polymers, exhibits unique characteristics that make it especially suitable for dynamic mechanical systems, including high stretchability, excellent elasticity, low-temperature flexibility, and superior resilience [11]. Since repeated mechanical loading is inherent to TENG-based energy harvesting, favorable dynamic mechanical properties are essential to ensure stable and uniform output performance. Consequently, various types of rubber materials have been employed as active components in TENGs [12,13,14,15,16,17,18]. Rubber-based TENGs have demonstrated considerable potential for applications in energy harvesting and self-powered sensing systems [17,18,19].

Recent studies have shown that the output performance of TENGs can be significantly enhanced by incorporating conductive fillers into dielectric materials [17,18,20,21]. Conductive fillers can effectively increase the dielectric constant of composite systems through the microcapacitor model, thereby enhancing the overall capacitance of the material [22]. In general, higher composite capacitance leads to stronger current and voltage outputs by sustaining greater electrical pressure during operation [17,18]. Additionally, improved electrical conductivity facilitates efficient charge extraction and transport, further contributing to enhanced triboelectric performance [17,18].

Conductive carbon nanomaterials are widely recognized as promising candidates for TENG applications owing to their excellent electrical conductivity, mechanical flexibility, and tunable surface properties. These advantages enable notable improvements in output performance, efficiency, and durability of TENG devices [23]. Nevertheless, carbon nanomaterials exhibit certain limitations, particularly in terms of magnetic functionality, filler dispersion and cost competitiveness compared to inexpensive metallic conductors [24,25,26]. Moreover, carbon nanofillers can form chemical bonds with rubber chains, which may restrict relative motion at the rubber–filler interface, thereby diminishing the effectiveness of triboelectrification [27]. Additionally, the increased elasticity associated with strong filler–polymer chemical bonding can reduce the fraction of applied mechanical energy converted into electrical output. On the other hand, ceramic materials can improve the dielectric properties of rubber composites. However, owing to the significant polarity mismatch between the filler and the rubber matrix, the fillers require surface modification to achieve improved TENG performance [17,18]. Consequently, conductive fillers that interact with rubber matrices through strong yet flexible physical interactions are more desirable for enhancing triboelectric performance. In this context, silver nanomaterials have recently been employed to improve TENG output characteristics [28,29,30]. However, the high cost of silver limits its practicality for large-scale energy harvesting applications.

Due to the presence of free electrons, metals are highly polarizable [31]. In the presence of an electric field, mobile electrons readily rearrange within the lattice, generating large induced dipole moments. As a result, metallic fillers can induce strong interfacial polarization at the filler–rubber interface, thereby enhancing the energy conversion efficiency of triboelectric rubber composites. However, interfacial polarization becomes more pronounced with stronger filler–rubber interactions. Alam et al. [32] demonstrated that the amount of generated triboelectric charge in rubber composites is directly proportional to the filler–rubber interaction energy. This interaction energy depends strongly on the compatibility between the functional groups of the rubber and the filler. It is noteworthy that although a metallic electrode and a non-conductive rubber interface can form a triboelectric pair, embedding conductive fillers within the rubber matrix significantly increases the interfacial contact area, resulting in higher triboelectric charge generation throughout the system [18]. Therefore, the uniform dispersion of conductive materials within dielectric polymers, combined with strong filler–polymer interactions, represents an effective strategy for improving triboelectric energy harvesting efficiency [32].

Various rubber–filler composites have been investigated for triboelectric nanogenerators (TENGs). Lu et al. [15] reported a carboxymethyl styrene–butadiene/barium titanate composite that delivered a power output of 6.14 W/m^2^, a transferred charge of 106 nC, and 211% stretchability. Bi et al. [33] developed a Cu-coated natural rubber TENG, achieving a maximum power density of 0.14 mW/m^2^ at 100% strain. Kim et al. [34] fabricated a silicone rubber/carbon nanotube (CNT) composite that exhibited a maximum power density of 3.52 W/m^2^ with an exceptional stretchability of 800%. Luu et al. [35] introduced a hydroxyapatite-loaded polydimethylsiloxane (PDMS) composite, achieving a surface charge density of 90.36 μC/m^2^, a power density of 27.34 W/m^2^, and stretchability of up to 290%.

The triboelectric performance of TENGs is strongly influenced by the physicochemical characteristics of both the rubber matrix and the filler because different rubber systems exhibit distinct interfacial interactions with different fillers. Consequently, the selection of an appropriate filler is crucial for maximizing the triboelectric performance of a specific rubber matrix. Among various elastomers, styrene–butadiene rubber (SBR) is particularly attractive for TENG applications owing to its excellent environmental stability, high concentration of π-electrons associated with the phenyl groups, enhanced electrical polarizability, and strong compatibility with a wide variety of functional fillers.

Alam et al. [17,18,32] investigated SBR-based TENGs incorporating hybrid filler systems comprising diatomaceous earth/CNTs, barium titanate/CNTs, and barium titanate/MoS_2_/CNTs. Their study demonstrated that these hybrid fillers reinforced the rubber matrix through strong interfacial interactions, resulting in enhanced triboelectric performance. Among the investigated systems, the barium titanate/MoS_2_/CNT hybrid exhibited the highest electrical output because MoS_2_ established strong σ–π intermolecular interactions with the SBR matrix. Furthermore, appreciable triboelectric charge generation was achieved even under a compressive strain of only 1%, indicating that efficient charge transfer can occur with minimal interfacial separation when strong rubber–filler interactions are present [32]. Although MoS_2_ significantly enhanced triboelectric charge generation, its relatively low electrical conductivity required the incorporation of conductive CNTs to facilitate efficient charge transport [32].

In this context, metallic fillers are particularly attractive because they can simultaneously function as triboelectric interfaces and electrically conductive pathways. Alam et al. [36,37] further demonstrated that iron particles exhibit strong interfacial interactions with SBR. Iron possesses intrinsic magnetic properties and forms strong interactions between its d-orbitals and the π-electrons of aromatic benzene rings [38,39]. Since the SBR backbone contains numerous phenyl groups, strong interactions with transition metals such as iron are anticipated. Because rubber–filler interfacial interactions play a pivotal role in triboelectric charge generation, metal–rubber interfaces may serve as highly effective triboelectric pairs with considerable energy-harvesting potential. Although metals generally possess high electrical and thermal conductivities, their interfacial interactions with rubber matrices vary depending on their chemical characteristics and surface functionalities [37].

Most previous studies have interpreted TENG performance primarily in terms of contact electrification and differences in electron affinity. However, the influence of reinforcement arising from intermolecular interfacial interactions has received comparatively little attention. A systematic investigation of the relationship between rubber–metal interfacial interactions, composite reinforcement, and triboelectric charge generation is therefore essential for elucidating the energy-harvesting mechanisms of metallic filler-reinforced rubber composites. To the best of our knowledge, no systematic study has yet established the correlation between metal–rubber reinforcing interactions and triboelectric charge generation in metallic filler-filled rubber composites.

In the present study, micron-sized aluminum and iron powders were selected as conductive fillers to fabricate SBR composites for triboelectric energy harvesting applications. Compared with nanoparticles, micron-sized fillers can be dispersed more uniformly within the rubber matrix while offering lower cost and easier processing. Moreover, aluminum and iron are naturally abundant, economically viable, and environmentally benign. Iron-filled rubber composites are also attractive for vibration damping applications, enabling multifunctional materials capable of simultaneously dissipating mechanical vibrations and harvesting mechanical energy [40,41,42]. Strong filler–rubber interfacial interactions are expected to increase frictional energy dissipation at the interface, thereby promoting enhanced triboelectric charge generation during repeated contact and separation. Accordingly, the triboelectric performance of aluminum- and iron-filled SBR composites was systematically evaluated using a single-electrode TENG configuration and correlated with their mechanical reinforcement characteristics. Furthermore, the triboelectric energy-harvesting capability of the iron-filled SBR composite under biomechanical hand-patting excitation was also investigated to demonstrate its practical applicability.

## 2. Materials and Methods

### 2.1. Materials

**Figure 1:**
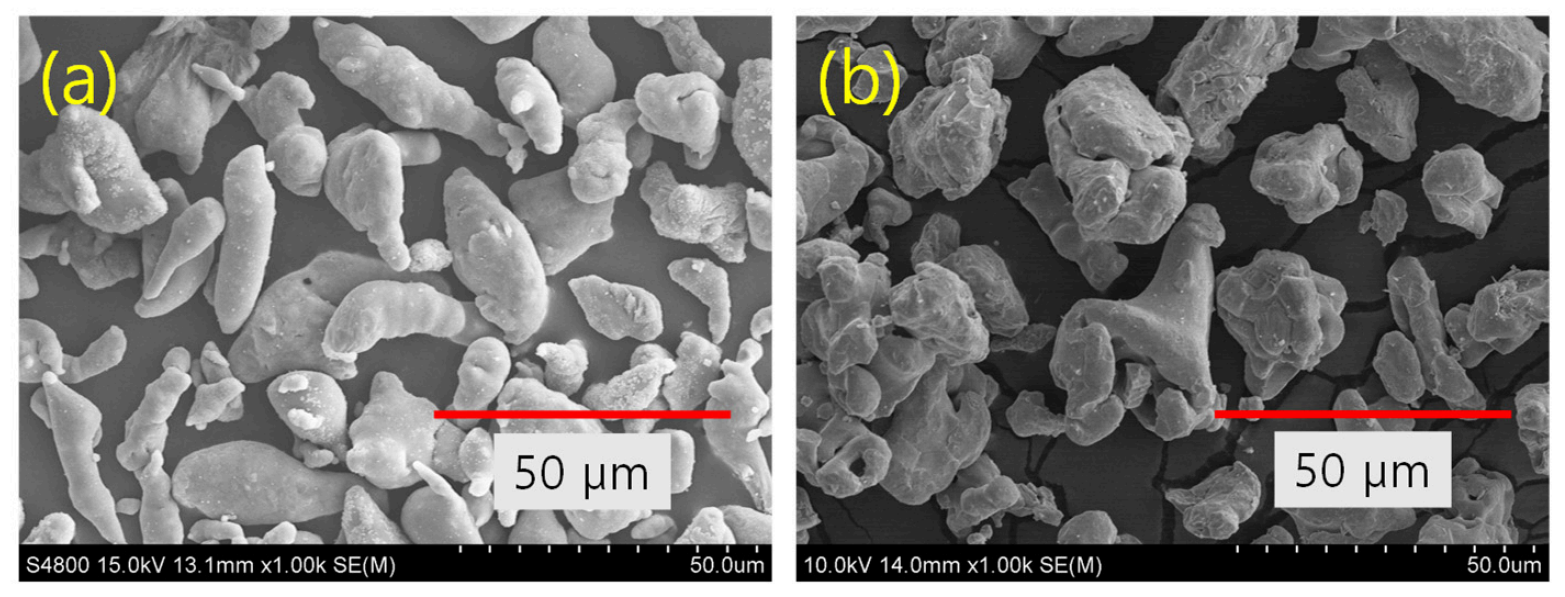
SEM images of metal powders: (**a**) aluminum and (**b**) iron.

Styrene–butadiene rubber (SBR 1502), containing 23.5% styrene monomer, was purchased from Kumho Petrochemicals (Seoul, Republic of Korea). A masterbatch formulation was prepared consisting of 100 g of SBR, 5 g of ZnO, 2 g of stearic acid, 1.75 g of N-tert-butylbenzothiazole sulfenamide (accelerator), 1 g of tetramethylthiuram disulfide (accelerator), and 1.5 g of sulfur. The masterbatch rubber was compounded using an open two-roll mill. Aluminum powder (average particle size: 325 mesh) was procured from Daejung Chemicals & Metals Co., Ltd. (Siheung-si, Republic of Korea), whereas electrolytic-grade iron powder (average particle size: 400 mesh) was supplied by AO Metal Corporation Ltd. (Hwaseong-si, Republic of Korea). Scanning electron microscopy (SEM) images of the metal powders are shown in Figure 1a,b, from which it is evident that the iron particles exhibit a more irregular morphology and slightly lower particle size compared to the aluminum particles. Such morphological differences may contribute to improved rubber reinforcement and enhanced filler–rubber frictional interactions in the Fe-filled composites.

Analytical-grade toluene was purchased from Daejung Chemicals & Metals Co., Ltd. (Siheung-si, Republic of Korea). All materials were used as received without further purification.

### 2.2. Fabrication of Rubber Composites

Aluminum- and iron-filled rubber composites containing 10–20 vol% fillers were prepared by a solvent-blending method to enhance filler–rubber interfacial adhesion, following the procedure reported previously [32]. Because the filler particles are several micrometers in size, relatively high filler loadings were employed to achieve the percolation threshold. Initially, 25 g of the rubber masterbatch was placed in a glass vessel, followed by the addition of 100 mL of toluene. The mixture was allowed to swell overnight to ensure complete solvent penetration into the rubber matrix. Subsequently, the swollen rubber was mechanically stirred to obtain a homogeneous slurry.

Separately, the required amount of filler (10–20 vol%, as listed in Table 1) was dispersed in 25 mL of toluene and sonicated for 30 min to achieve homogeneous dispersion. The filler dispersion was subsequently combined with the rubber slurry and mixed vigorously using a hand mixer for approximately 10 min. After thorough mixing, the compounded slurry was poured into a flat tray and dried in an oven at 80 °C for 24 h to remove the solvent.

The resulting filler-loaded rubber compounds were then vulcanized at 150 °C for 15 min using appropriate molds in a compression molding machine, as described elsewhere [37].

### 2.3. Measurement of Mechanical Properties

The compressive and tensile mechanical properties of the rubber composites were measured using a universal testing machine (UTM, Lloyd, Westminster, UK) equipped with a 1 kN load cell. For compressive testing, cylindrical specimens with a diameter of 20 mm and a height of 10 mm were used, and a constant compression rate of 2 mm/min was applied. Tensile properties were evaluated using dumbbell-shaped specimens with a gauge length of 25 mm, prepared according to ISO 37 (Type 2) [43], and tested at a crosshead speed of 300 mm/min. For each mechanical property, four independent tests were conducted, and the average values are reported. For stress relaxation measurements, cylindrical samples were compressed to 20% strain, and the relaxed stress values were measured for up to 60 s.

### 2.4. Temperature Sweep Dynamic Mechanical Analysis

Dynamic mechanical analysis (DMA) was performed using a dynamic mechanical analyzer (DMA Q800, TA Instruments, New Castle, DE, USA) in tensile mode over a temperature range of −50 to 80 °C at a heating rate of 2 °C min^−1^ under a nitrogen atmosphere. Prior to each measurement, the specimen was equilibrated at the initial test temperature for 5 min under isothermal conditions to ensure complete thermal stabilization. Rectangular specimens with dimensions of 10 mm × 4 mm × 1 mm were punched from the vulcanized rubber sheets and used for DMA characterization.

### 2.5. Measurement of Electrical and Triboelectric Properties Under Mechanical Loads

The electrical and triboelectric properties were measured using a digital multimeter (Keysight 34461A, Keysight Technologies, Santa Rosa, CA, USA). Triboelectric measurements were conducted in a single-electrode mode with a 10 MΩ input impedance at room temperature and 50% relative humidity. Cylindrical specimens (diameter = 20 mm, height = 10 mm) were subjected to uniaxial cyclic compressive strains of 1, 2, 3, 4, and 5% using a universal testing machine (UTM) at a frequency of approximately 0.85 Hz.

During compression, frictional interactions at the filler–rubber interfaces were assumed to generate triboelectric charges. Although the resulting interfacial deformation is relatively small, significant charge generation can still occur depending on the strength of filler–rubber interactions and the number of interfacial contacts within the composite.

The detailed experimental setup for triboelectric nanogenerator (TENG) measurements under mechanical deformation is shown in Figure S1 in the Supporting Information. Triboelectric performance metrics—including peak power density, peak current density, and total generated charge per cycle—were evaluated using the relevant equations provided in the Supporting Information.

### 2.6. X-Ray Photoelectron Spectroscopic Studies

X-ray photoelectron spectroscopy (XPS) was performed using an ESCALAB 250 spectrometer (Thermo Fisher Scientific, Loughborough, UK) equipped with a monochromatic Al Kα X-ray source (hν = 1486.6 eV) and a 400 μm beam spot size.

### 2.7. Raman Spectroscopy of Rubber Compounds

Raman spectroscopic analysis of the sheeted rubber compounds was carried out using a Raman spectrometer (Horiba Scientific, Kyoto, Japan) equipped with a 532 nm laser.

## 3. Results and Discussion

### 3.1. Mechanical Properties of Rubber Composites

**Figure 2:**
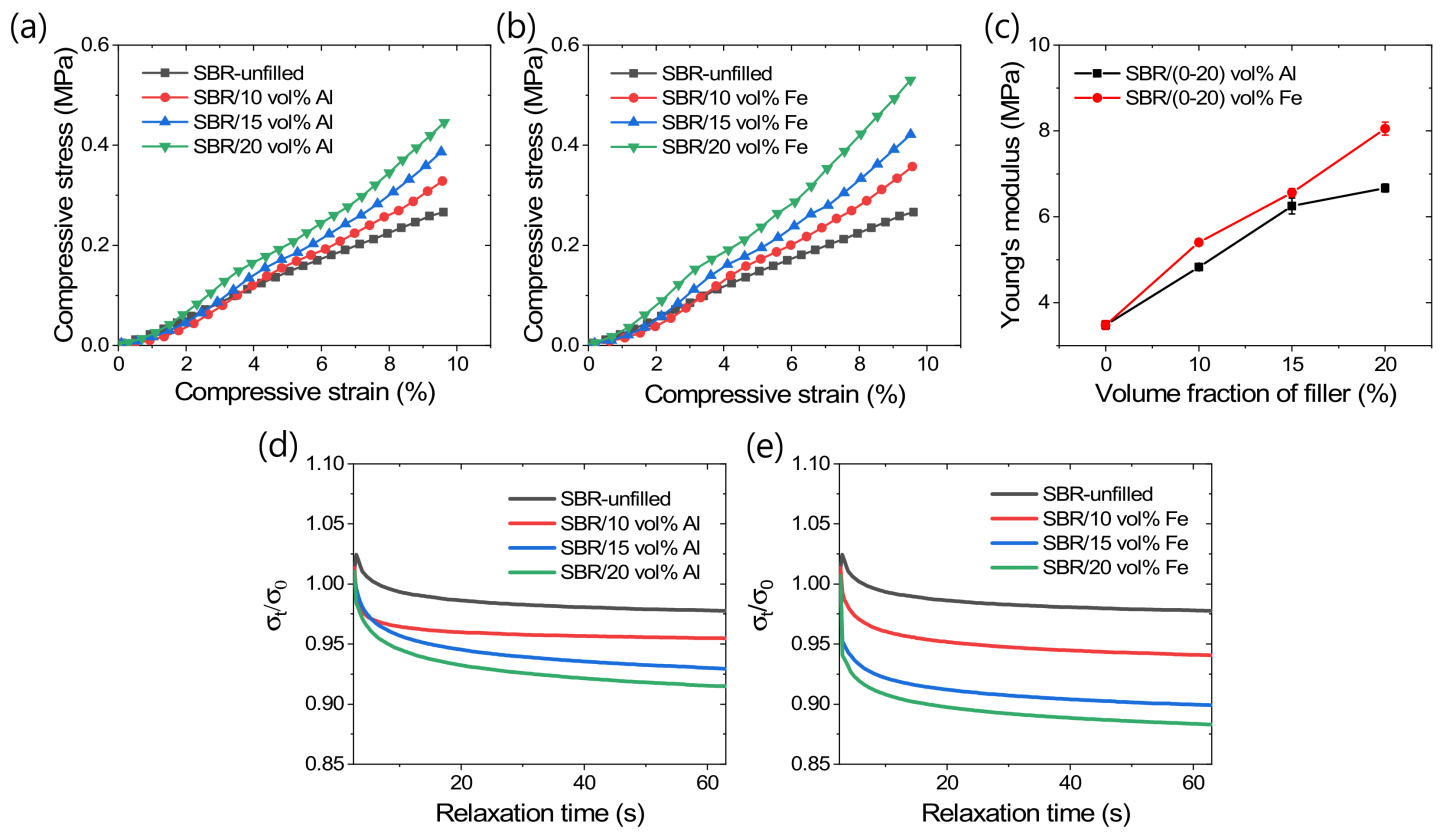
Compressive mechanical properties of rubber composites: (**a**) stress–strain curves of Al-filled rubber composites, (**b**) stress–strain curves of Fe-filled rubber composites, (**c**) Young’s modulus, (**d**) stress relaxation of Al-filled rubber composites, (**e**) stress relaxation of Fe-filled rubber composites.

The compressive mechanical properties of the rubber composites are presented in Figure 2a–c and Figure S2a–d. From the load–compressive deformation curves shown in Figure S2a–d, it is observed that the work done on the composites increases markedly beyond a deformation of 0.1 mm and becomes more than twice as large upon doubling the compressive strain.

As evident from Figure 2a,b, the compressive stress of the rubber composites increases progressively with increasing compressive strain. However, the stress–strain slopes are not uniform across the entire strain range. Specifically, the slope increases up to approximately 4–5% strain, decreases slightly in the intermediate strain range of 5–7%, and subsequently increases again at higher strains. Furthermore, Figure 2a,b indicate that increasing filler content enhances the strain dependence of the modulus, particularly in the low-strain region. This behavior can be attributed to strain-dependent changes in interfacial adhesion and filler interactions.

At the initial stage, filler particles are separated by relatively large distances, and filler–filler mechanical interactions are minimal. As the compressive strain increases, the interparticle distance decreases, leading to enhanced mechanical interactions among filler particles and possible slippage of loosely bound fillers, which reduces the stress–strain slope in the intermediate strain regime. With further increases in strain, such slippage is minimized or suppressed, and the formation of more continuous and robust filler networks may occur, resulting in a renewed increase in the stress–strain slope.

Additionally, Figure 2b, together with the Young’s modulus values shown in Figure 2c, indicates that Fe-filled composites exhibit a stronger reinforcing effect than Al-filled composites at comparable filler volume fractions. Although Fe, as a transition (d-block) metal, is known to exert a greater chemical influence on reducing the cross-link density of rubber composites compared to p-block metals such as Al—which would typically be expected to reduce compressive strength—this trend is not observed in the present system [37]. The Fe particles are smaller in size and possess a higher filler structure than the Al particles. Owing to these physical characteristics, Fe particles provide more effective physical reinforcement of the rubber matrix than Al particles, despite the lower cross-link density of the Fe-filled composites. The stress-relaxation results presented in Figure 2d,e further support a predominantly physical reinforcement mechanism rather than a chemical one. A comparison of Figure 2d,e reveals that the Fe-filled composites exhibit higher stress-relaxation than the Al-filled composites. In contrast, the unfilled rubber shows the lowest stress-relaxation because its network is dominated by covalent cross-links with minimal polymer–filler physical interactions [37]. Notably, the stress-relaxation increases markedly with filler loading up to 15 vol%, followed by only a slight increase at higher filler contents, suggesting that the filler–rubber interfacial interactions approach saturation beyond this concentration.

Although Fe slightly reduces the cross-link density, its strong interfacial interactions with the SBR matrix, arising primarily from electrostatic interactions and d–π interactions between Fe atoms and the phenyl groups of the cross-linked polymer chains, dominate the mechanical response [36,37,38,39]. These interactions are sufficiently strong to compensate for the loss of cross-links, resulting in superior compressive mechanical properties for Fe-based composites compared to Al-based composites, particularly when SBR is used as the rubber matrix. Moreover, the irregular morphology of Fe fillers is expected to contribute only marginally to the enhancement of the compressive modulus [44].

**Figure 3:**
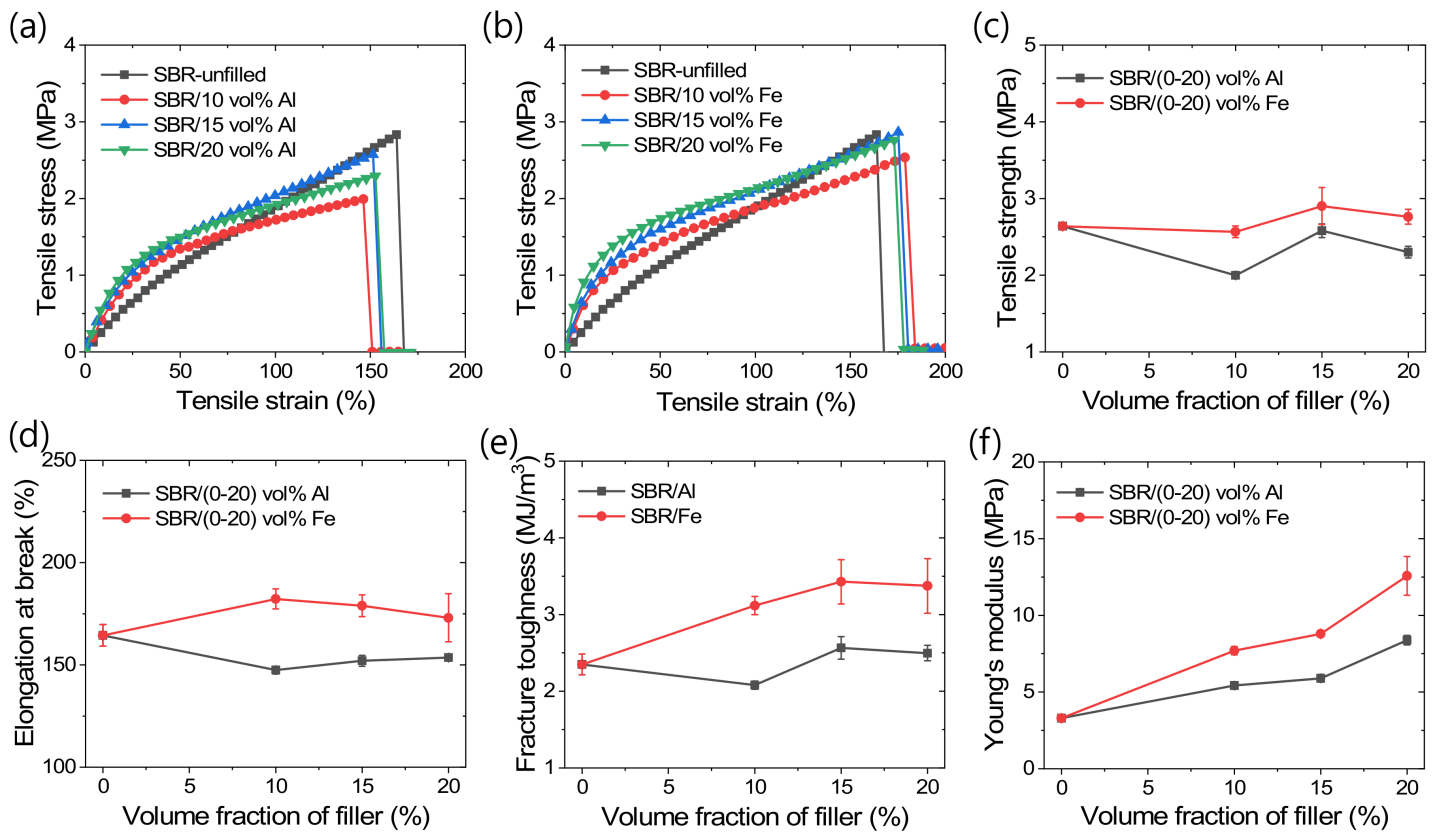
Tensile mechanical properties of rubber composites: (**a**) stress–strain curves of Al-filled rubber composites, (**b**) stress–strain curves of Fe-filled rubber composites, (**c**) tensile strength, (**d**) elongation at break, (**e**) fracture toughness, and (**f**) Young’s modulus.

The tensile mechanical properties of the rubber composites are presented in Figure 3a–f. From the stress–strain curves shown in Figure 3a,b, it is evident that both Al- and Fe-based rubber composites exhibit similar overall deformation behavior. At low strain levels, the stress–strain slope is highest; however, with increasing strain, the slope gradually decreases and becomes nearly constant beyond a critical strain. This tensile response suggests that the contribution of filler networks to the tensile modulus becomes negligible beyond this critical strain.

In terms of tensile modulus, Fe-based composites exhibit higher values at low strains, indicating the formation of stronger filler networks compared to Al-based rubber composites at similar filler volume fractions [44]. The tensile strength data presented in Figure 3c further demonstrate that Fe particles establish stronger physical interactions with the rubber chains, leading to enhanced tensile strength relative to Al-filled composites.

In contrast, despite a partial reduction in cross-link density, the stronger interfacial interactions associated with Fe fillers [37] result in significantly higher stretchability of Fe-based rubber composites, as evidenced by the elongation-at-break values shown in Figure 3d. Owing to the simultaneous improvement in tensile strength and elongation at break, the fracture toughness of Fe-based rubber composites is markedly higher than that of Al-based composites (Figure 3e).

Notably, despite the micrometer-sized filler particles, the incorporation of an optimal filler loading results in slight improvements in tensile strength, elongation at break, and fracture toughness. These enhancements indicate the presence of effective filler–rubber physical interactions that compensate for, and partially overcome, the reduction in mechanical strength associated with the lower cross-link density compared with the unfilled rubber. Furthermore, the tensile Young’s modulus values shown in Figure 3f suggest that the filler network becomes significantly strengthened beyond a filler content of 15 vol%.

**Figure 4:**
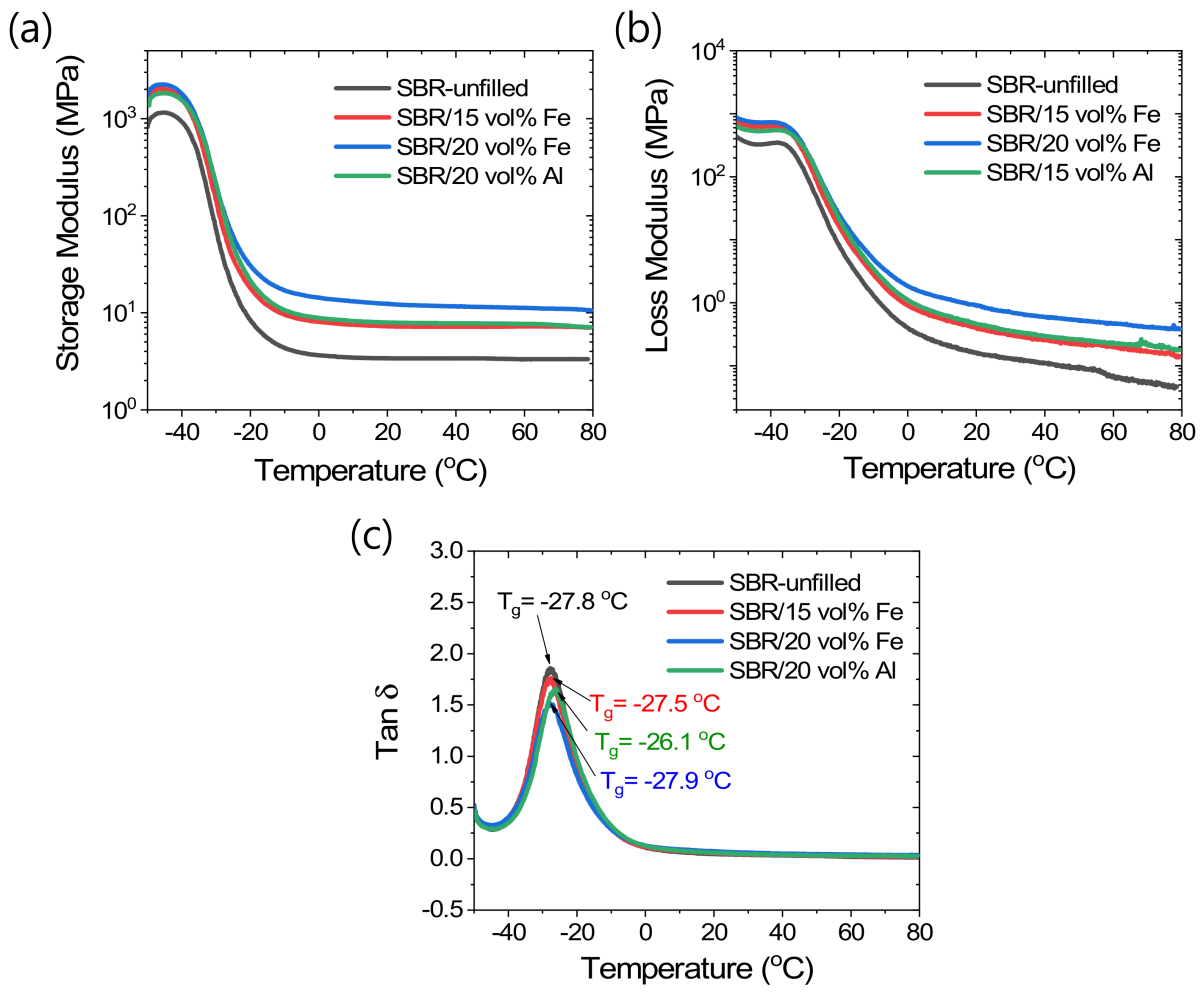
Temperature-dependent dynamic mechanical properties of rubber composites: (**a**) storage modulus, (**b**) loss modulus, and (**c**) tan δ curves.

The dynamic mechanical properties of the rubber composites are presented in Figure 4a–c. As shown in Figure 4a, the storage modulus increases with increasing filler content across the entire temperature range, which can be attributed to the incorporation of rigid filler structures within the rubber matrix and the resulting restriction of polymer chain mobility. Upon increasing the temperature from the glassy region, the storage modulus decreases significantly due to the transition from the glassy state to the rubbery state associated with increased segmental mobility. Beyond the critical transition region near 0 °C, all rubber composites exhibit a relatively stable rubbery plateau up to the maximum measurement temperature. Notably, the reinforcing effect of 15 vol% Fe filler is comparable to that of 20 vol% Al filler, indicating the higher reinforcing efficiency of Fe particles in the SBR matrix.

The loss modulus exhibits a similar dependence on filler type and loading, as shown in Figure 4b. However, unlike the storage modulus, the loss modulus continuously decreases with increasing temperature. Since the loss modulus reflects energy dissipation associated with polymer–filler interfacial interactions and molecular friction, its continuous reduction with temperature suggests a weakening of filler–rubber interactions due to enhanced polymer chain mobility at elevated temperatures.

The temperature-dependent tan δ curves are presented in Figure 4c. The decrease in tan δ peak intensity with increasing filler content indicates enhanced restriction of polymer chain motion and improved composite reinforcement. Interestingly, Al-filled composites exhibit a slight shift in the glass transition temperature (T_g_) toward higher temperatures, whereas Fe-filled composites maintain T_g_ values comparable to those of unfilled SBR. Previous studies have reported that metallic fillers, including Al and Fe, can reduce the cross-link density of SBR composites, with Fe-filled systems showing a more pronounced reduction [37]. Therefore, the slight increase in T_g_ observed for Al-filled composites may originate from the greater contribution of rigid filler structures, which restrict segmental motion despite the reduced cross-link density. In contrast, Fe-filled composites achieve a balance between structural rigidity and chain flexibility, where the reinforcing effect of a higher filler structure compensates for the increased flexibility associated with the lower cross-link density.

Based on the aforementioned mechanical evaluations, Fe-filled rubber composites exhibit significantly improved viscoelastic properties compared with Al-filled rubber composites. Overall, the 15 vol% Fe-filled composite demonstrates the most favorable balance of tensile strength, fracture toughness, stretchability, and dynamic mechanical performance, highlighting its potential as a high-performance stretchable material for triboelectric nanogenerator (TENG) applications.

**Figure 5:**
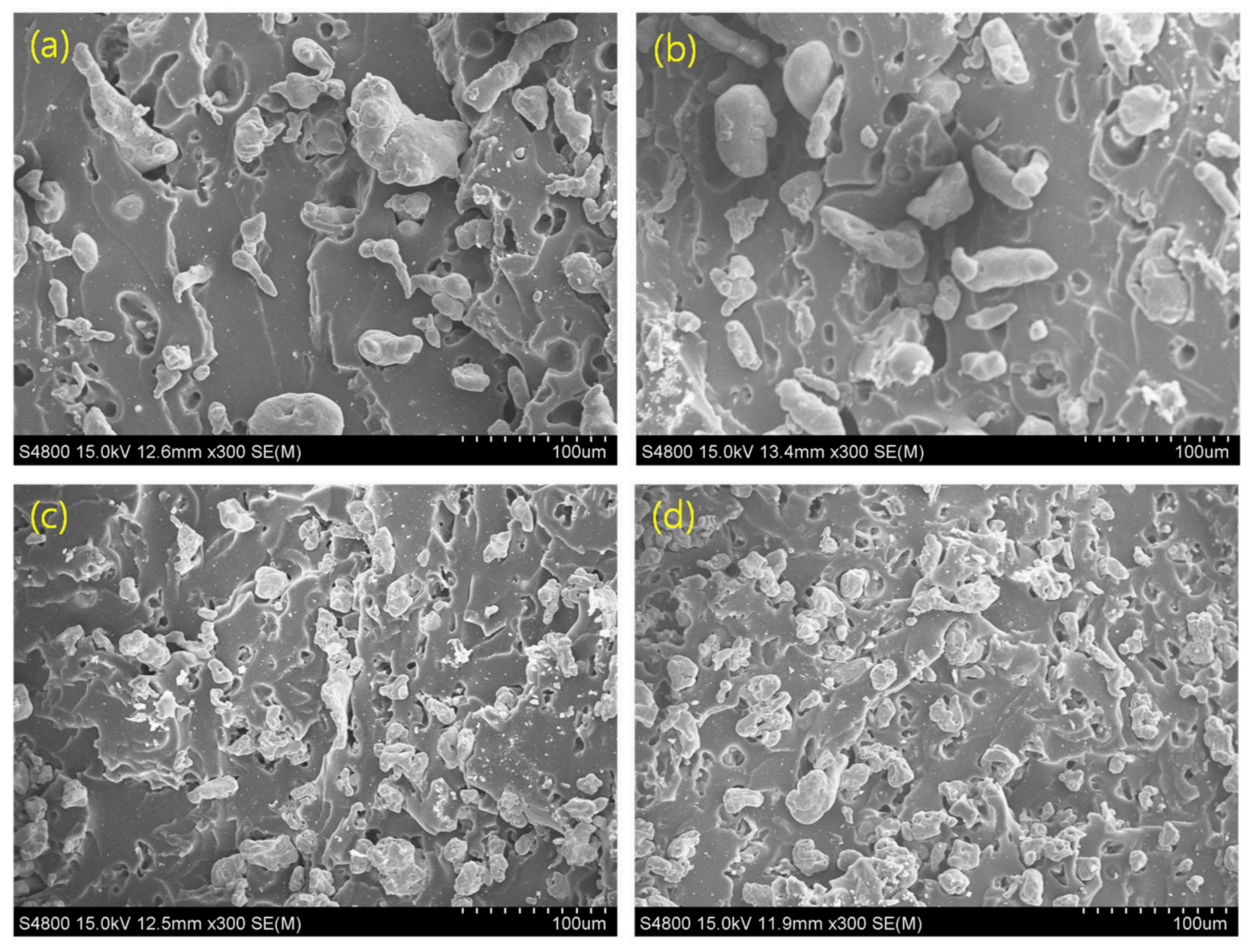
SEM images of rubber composites: (**a**) SBR/15 vol% Al, (**b**) SBR/20 vol% Al, (**c**) SBR/15 vol% Fe, and (**d**) SBR/20 vol% Fe.

Scanning electron microscopy (SEM) images of the fractured surfaces of the rubber composites are presented in Figure 5a–d. A comparison of Figure 5a,b with Figure 5c,d clearly indicates that Fe particles exhibit stronger adhesion to the rubber matrix than Al particles. However, the Fe-filled composite containing 20 vol% filler exhibits significant filler aggregation, as observed in Figure 5d. In contrast, the 15 vol% Fe-filled rubber composite demonstrates a well-developed filler network with a more uniform and homogeneous filler dispersion, as shown in Figure 5c.

These morphological observations suggest that Fe particles interact more strongly with the SBR matrix, likely through interfacial interactions [36,37,38]. As a result of the stronger interfacial interactions, the interparticle distances in Fe-filled composites are reduced compared to those in Al-filled composites.

In contrast, the relatively weaker interactions between Al particles and the rubber matrix may lead to the formation of dielectric gaps at the particle–matrix interface, thereby imparting a more non-conductive character to the Al-based composites. Conversely, the strong adhesion between Fe particles and the rubber matrix promotes the formation of interconnected conductive pathways, which is consistent with the enhanced electrical conductivity observed for Fe-based composites in the electrical property measurements discussed in a later section.

### 3.2. Electrical Properties of Rubber Composites

**Figure 6:**
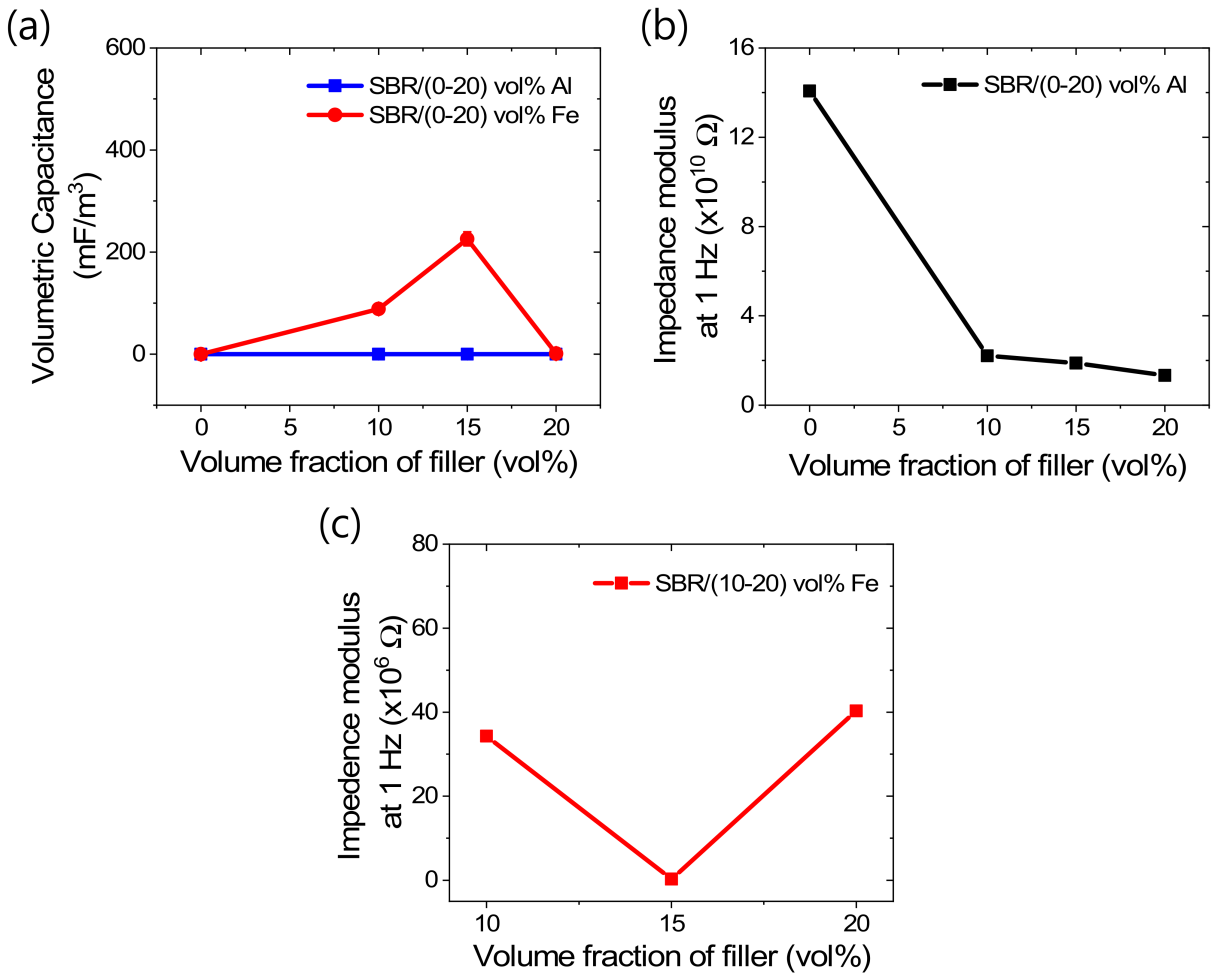
Electrical properties of rubber composites: (**a**) volumetric capacitance, (**b**) impedance modulus of SBR/(0–20) vol% Al composites, and (**c**) impedance modulus of SBR/(10–20) vol% Fe composites.

The electrical properties of the rubber composites are presented in Figure 6a–c and Figure S3a–d. Figure 6a and Figure S3a,b illustrate the volumetric capacitance and measured capacitance values of the cylindrical rubber composites. It is evident that Fe-filled rubber composites exhibit significantly higher capacitance values compared with both unfilled and Al-filled rubber composites. Notably, the volumetric capacitance of the Fe-filled composites increases with increasing filler loading up to 15 vol% and subsequently decreases substantially at higher filler concentrations.

It is well established that the incorporation of conductive fillers into dielectric matrices near the percolation threshold promotes the formation of numerous microcapacitor networks, thereby enhancing the overall capacitance according to the microcapacitor model [45,46,47]. However, further increases in filler loading can promote filler aggregation, as observed in the SEM images, resulting in reduced effective interfacial polarization and lower capacitance. Due to the limited electrical conductivity and poor conductive network formation in Al-filled rubber composites, their capacitance values remain considerably lower. Although high capacitance alone does not directly determine triboelectric charge generation, it can reduce the electrical impedance modulus, improve charge accumulation, and facilitate efficient power transfer to the external electrode.

Figure 6b,c and Figure S3c,d show the variation in electrical impedance modulus and resistance as a function of filler volume fraction. Fe-filled rubber composites exhibit a pronounced decrease in both impedance modulus and electrical resistance, with a sharp transition observed at a filler loading of 15 vol%. Further increases in filler concentration result in only marginal reductions in resistance but a subsequent increase in impedance modulus. This behavior indicates that capacitive contributions significantly influence the alternating current (AC) conductivity of the composites. Beyond the percolation threshold, excessive filler loading leads to particle aggregation and weaker filler–rubber interfacial interactions, which disrupt effective conductive pathways, reduce capacitance, and consequently increase the impedance modulus.

The resistance behavior suggests that electrical transport in the rubber composites occurs not only through direct particle-to-particle contact but also via electron tunneling mechanisms [48,49,50]. At low filler contents, the tunneling contribution is minimal due to large interparticle distances; however, at an optimal filler loading, tunneling pathways become saturated, and additional filler content produces only a slight enhancement in tunneling conduction. In contrast, for Al-based rubber composites, the weak interfacial adhesion between Al particles and the rubber matrix, as evidenced by SEM observations, likely introduces dielectric barriers at the filler–matrix interfaces. These barriers suppress electron tunneling, resulting in higher resistance and impedance modulus.

Electrical impedance modulus is a critical parameter in triboelectric energy harvesting because it governs the efficient transfer of triboelectrically generated charges. A large impedance mismatch with input impedance can cause the generated charges to remain localized as static charges, resulting in their dissipation primarily as heat and consequently reducing the overall energy conversion efficiency.

### 3.3. Energy Harvesting Through Mechanical Deformation of Rubber Composites

**Figure 7:**
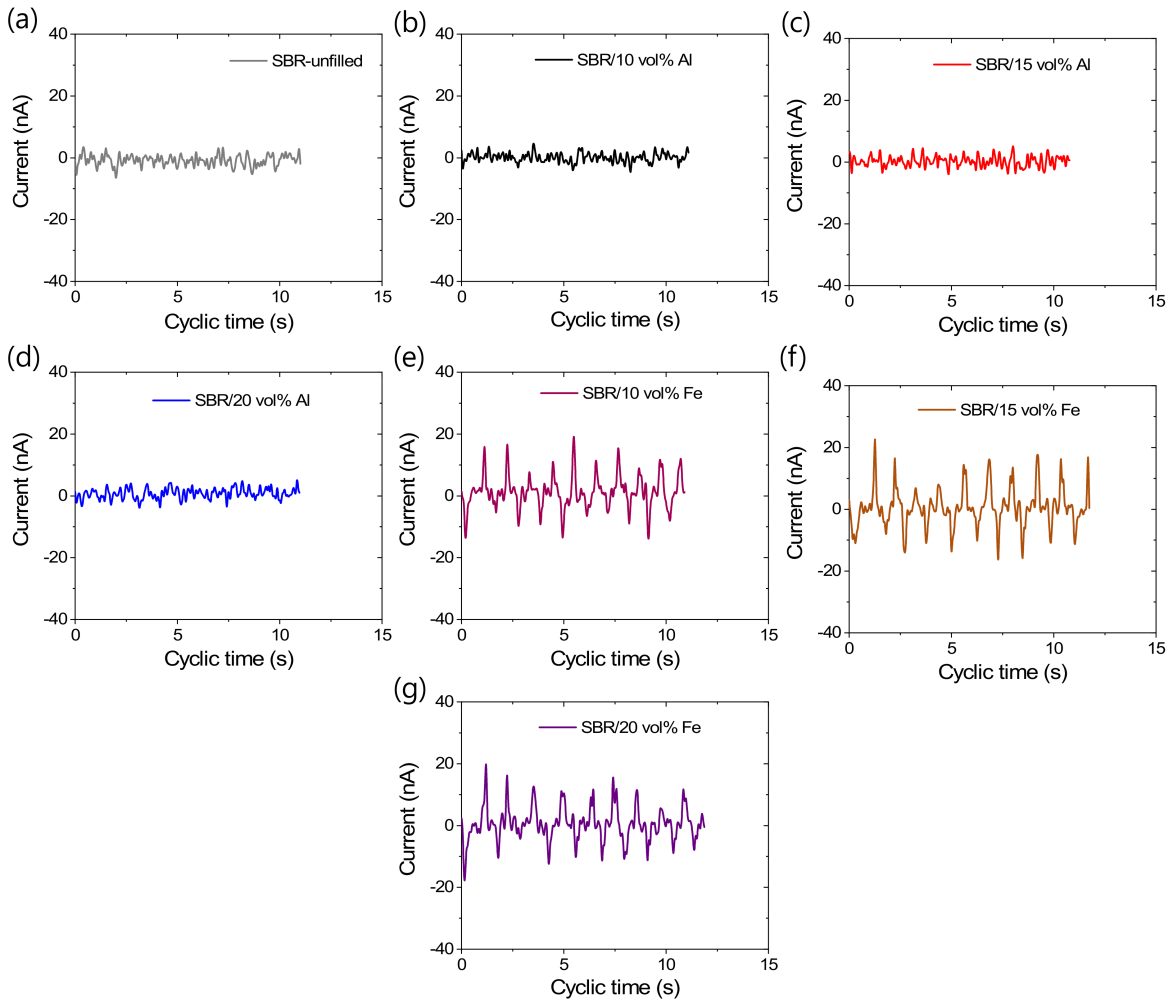
Triboelectric current patterns at 1% compressive strain cycles for different rubber composites: (**a**) SBR-unfilled, (**b**) SBR/10 vol% Al, (**c**) SBR/15 vol% Al, (**d**) SBR/20 vol% Al, (**e**) SBR/10 vol% Fe, (**f**) SBR/15 vol% Fe, and (**g**) SBR/20 vol% Fe.

The triboelectric current responses of the rubber composites under 1% compressive strain (corresponding to 0.1 mm cyclic deformation) are presented in Figure 7a–g. As evident from these figures, the Fe-based rubber composites exhibit markedly higher peak current intensities than the Al-based composites. This enhancement can be attributed to stronger filler–rubber interfacial interactions and higher electrical conductivity in the Fe-filled composites, which facilitate efficient extraction and transport of triboelectrically generated charges throughout the material. In contrast, the insufficient electrical conductivity of the Al-based rubber composites results in substantially lower current outputs.

Negative current peaks are observed during the compression phase, while positive current peaks appear during force release, as clearly shown in Figure 7e–g. These current profiles indicate that composites with higher electrical conductivity generate significantly greater triboelectric current intensities. For the unfilled SBR and Al-based rubber composites shown in Figure 7a–d, the current output increases only slightly with increasing filler content, which may be attributed to a marginal improvement in conductivity; however, it remains insufficient for effective charge collection and transport. Conversely, Fe-based rubber composites exhibit pronounced current generation owing to their substantial electrical conductivity and strong filler–rubber interfacial interactions.

Interestingly, although increased conductivity generally enhances peak current output, this effect is limited to a specific range of conductivity and filler loading. As shown in Figure 7e–g, the current intensity increases with Fe content up to 15 vol% and then decreases at higher filler loadings. Triboelectric charge generation is governed by both filler–matrix and matrix–counter-electrode interfacial interactions, with stronger interfacial interactions typically leading to higher charge generation. Based on the observed current outputs and corresponding mechanical properties, the maximum effective triboelectric interfacial area appears to occur at approximately 15 vol% Fe loading.

Beyond this optimal filler content, increased filler–filler networking reduces the available filler–rubber triboelectric interfaces, leading to diminished charge generation and lower current output. This trend is further supported by the observation that the 10 vol% Fe composite exhibits current intensities comparable to those of the 20 vol% Fe composite (Figure 7e,g). Therefore, at filler contents exceeding the optimal level, the reduction in effective filler–rubber triboelectric interfaces outweighs the benefits of increased conductivity, resulting in decreased triboelectric current output.

**Figure 8:**
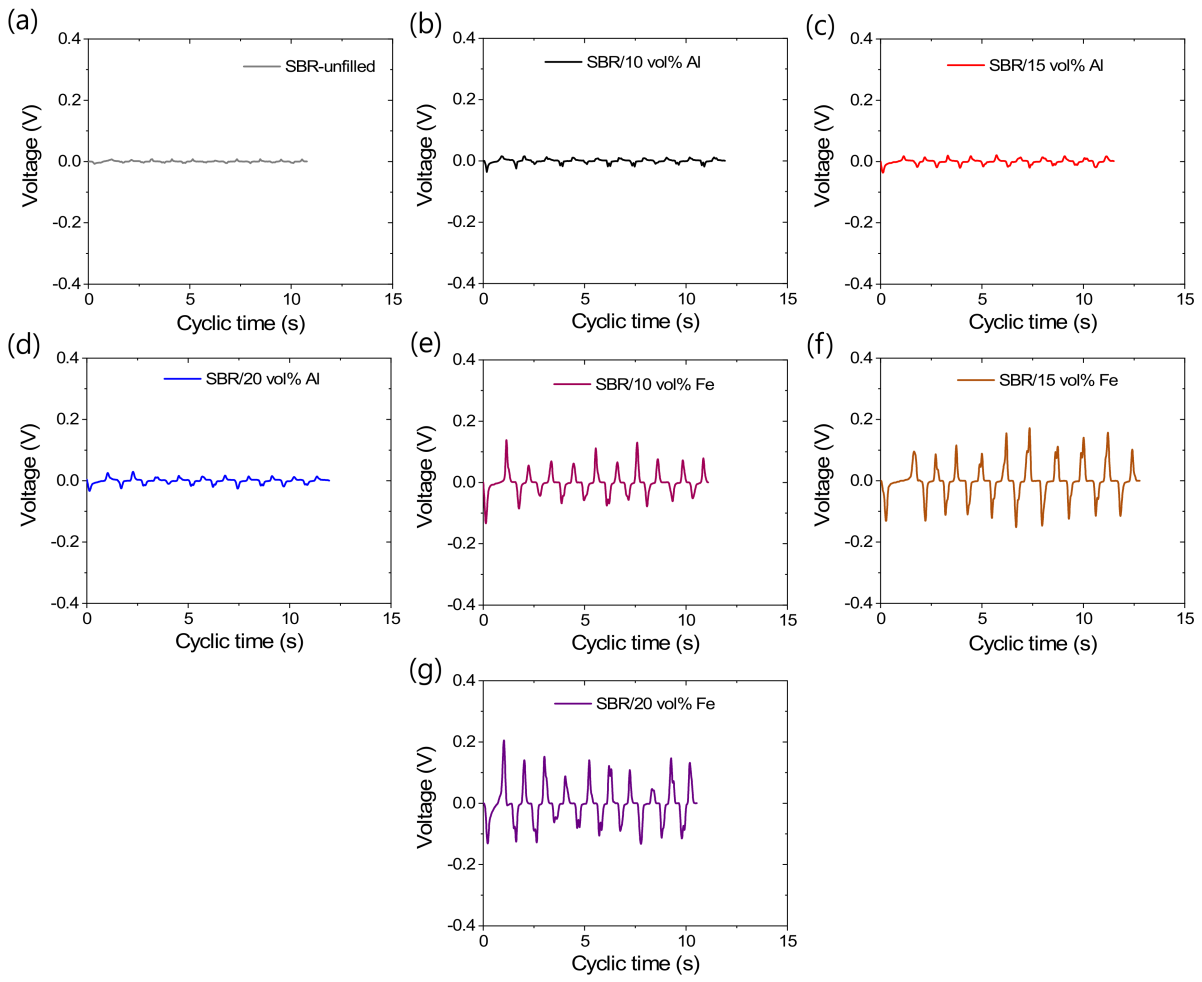
Triboelectric voltage patterns at 1% compressive strain cycles for different rubber composites: (**a**) SBR-unfilled, (**b**) SBR/10 vol% Al, (**c**) SBR/15 vol% Al, (**d**) SBR/20 vol% Al, (**e**) SBR/10 vol% Fe, (**f**) SBR/15 vol% Fe, and (**g**) SBR/20 vol% Fe.

The triboelectric voltage responses of the rubber composites under 1% cyclic compressive strain are presented in Figure 8a–g. Consistent with the current output behavior, the Al-based rubber composites exhibit substantially lower voltage outputs than the Fe-based composites. As shown in Figure 8a–d, the voltage output of the unfilled SBR and Al-filled composites remains nearly unchanged with increasing filler content. In contrast, the Fe-based rubber composites in Figure 8e–g display significantly higher voltage outputs.

Further examination of Figure 8e–g reveals that the Fe-filled composite with 10 vol% Fe exhibits higher current intensity but lower voltage output compared to the 20 vol% Fe composite, which shows lower current intensity but higher voltage output. Notably, the composite containing 15 vol% Fe displays the highest intensities in both current and voltage outputs, despite also exhibiting the highest capacitance. These observations suggest that while capacitance is useful for identifying filler percolation behavior, it alone cannot reliably predict the triboelectric current or voltage output of nanogenerators. However, the impedance modulus can be an important factor influencing the triboelectric output performance. In this regard, unfilled and Al-filled rubber composites exhibit significantly higher impedance moduli compared with Fe-filled rubber composites. Consequently, Fe-filled rubber composites demonstrate enhanced electrical output signals due to their lower impedance and improved charge transfer capability. Among the investigated compositions, the 15 vol% Fe-filled composite exhibits the lowest impedance modulus along with the strongest filler–rubber interfacial interactions, which is expected to contribute to the highest current and voltage output signals.

At an optimal conductive filler loading, the composite capacitance increases significantly due to an enhanced interfacial polarization and reduced distance between conductive particles [45,46,47], while the number of active triboelectric interfaces is simultaneously maximized. This results in the generation of a higher density of electrical charges and, consequently, stronger current and voltage outputs. Therefore, achieving superior triboelectric performance requires a synergistic balance between high electrical conductivity, enhanced capacitance, and lower impedance within the rubber composite.

Near the filler percolation threshold, the rubber composites become highly sensitive to mechanical deformation, making them suitable for mechanical sensing applications. Moreover, because the composites exhibit more uniform and stable voltage and current responses at or above the percolation threshold, they are particularly promising as reliable electromechanical sensors [17,18].

**Figure 9:**
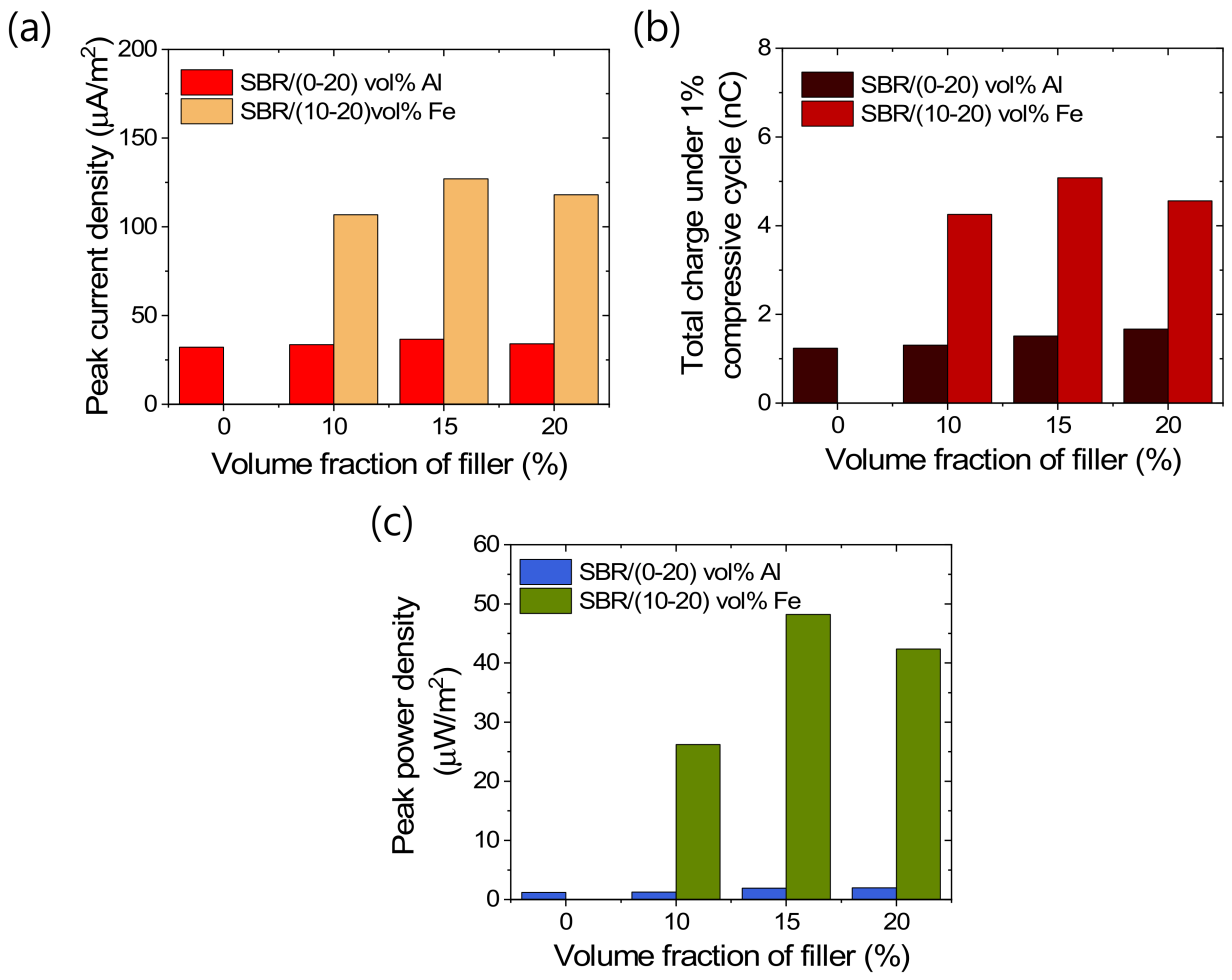
Triboelectric parameters of rubber composites with different filler volume fractions: (**a**) peak current density, (**b**) total charge under 1% compressive cycle, and (**c**) peak power density.

The triboelectric energy-harvesting efficiencies of the rubber composites under 1% cyclic compressive strain are presented in Figure 9a–c. As shown in Figure 9a, the peak current densities of Fe-based rubber composites are substantially higher than those of unfilled rubber and Al-based composites across all filler contents, with the maximum value observed for the composite containing 15 vol% Fe. Similarly, Figure 9b shows that Fe-based rubber composites generate significantly higher charges than Al-based composites, with the highest value again obtained at 15 vol% Fe loading.

Figure 9c further reveals pronounced differences in peak power densities between Al- and Fe-based rubber composites. This disparity arises from the considerably lower current and voltage outputs of the Al-based composites compared to their Fe-based counterparts at all filler concentrations. High power density is a critical parameter for self-powered sensing electrodes, as it ensures strong and uniform triboelectric voltage signals required for accurate force recognition and pattern sensing [17,18].

The superior peak current density and total charge under a cycle of Fe-based rubber composites can be attributed to stronger filler–rubber interfacial interactions [37] and enhanced electrical conductivity. Since triboelectric charge generation is intrinsically linked to filler–rubber interfacial interactions, an optimal filler loading is required to maximize the number of effective triboelectric pairs. At filler contents below or above this critical concentration, the effective interfacial area is reduced due to the lower number of triboelectric pairs or excessive filler–filler networking, respectively, leading to diminished triboelectric efficiency.

Consequently, the rubber composite containing 15 vol% Fe exhibits optimal performance, delivering the highest peak current density of 127.05 µA m^−2^, the highest generated charge of 5.01 nC, and the highest peak power density of 48.22 µW m^−2^ at 1% compressive strain. These values correspond to enhancements of 246%, 236%, and 2398%, respectively, compared to the Al-filled rubber composite at the same filler loading.

Since triboelectric charge generation in the rubber composites is driven by mechanical deformation, voltage outputs were examined under increasing deformation amplitudes, as shown in Figure S4a–g. These results demonstrate that increasing the compressive deformation amplitude leads to a pronounced increase in voltage output up to approximately 4% compressive strain, beyond which no significant enhancement is observed. This initial improvement can be attributed to increased relative motion at the rubber–filler interfaces, which promotes greater triboelectric charge generation.

At higher deformation amplitudes, however, the prolonged deformation time facilitates charge relaxation, thereby limiting further increases in voltage output beyond 4% strain. Notably, a comparison of Figure S4a–d and Figure S4e–g reveals that the voltage increment becomes more pronounced with increasing Fe filler content. This behavior can be attributed to the stronger filler–rubber interfacial interactions in Fe-filled composites, as evidenced by their higher stress-relaxation and loss modulus values. These enhanced interfacial interactions promote relative interfacial motion and facilitate more efficient mechanical energy dissipation during deformation [37].

Although larger deformation amplitudes result in higher voltage outputs, the corresponding mechanical work required does not scale proportionally. During compression, mechanical work must overcome the elastic resistance of the composite. In this system, the mechanical work more than doubles when the deformation strain is doubled (Figure S2b,d), whereas the voltage output increases by less than a factor of two. Consequently, from an energy conversion standpoint, smaller deformation amplitudes are more favorable for achieving higher mechanical work-to-electrical output efficiency.

Table 2 presents the mechanical load-to-charge generation efficiencies of the different rubber composites under 1% compressive strain. The results indicate that unfilled rubber and Al-based composites exhibit significantly lower charge generation efficiencies than Fe-based rubber composites under the same mechanical load. The highest charge generation efficiency, 0.927 nC/N, is obtained for the composite containing 15 vol% Fe.

Ramesh et al. [51] reviewed materials with very high piezoelectric coefficients. In comparison, the obtained value of 0.927 nC/N is competitive with, and in some cases exceeds, those of many commonly used piezoelectric materials. Additionally, rubber composites are inherently soft and stretchable, making them well suited for energy harvesting in ambient mechanical environments.

In addition to triboelectric performance, durability and fatigue resistance are critical parameters for the practical implementation of TENG devices. Based on the preceding analyses, the SBR composite containing 15 vol% Fe emerges as the optimal formulation in terms of both mechanical robustness and triboelectric output. Furthermore, compressive mechanical measurements indicate that the rubber composites can be repeatedly deformed up to 3% strain without adverse effects on elasticity, as evidenced by the stable stress–strain response within this deformation range. It should be noted that, at such low compressive strain levels, the rubber composites can withstand thousands of loading cycles without failure, demonstrating excellent mechanical resilience.

**Figure 10:**
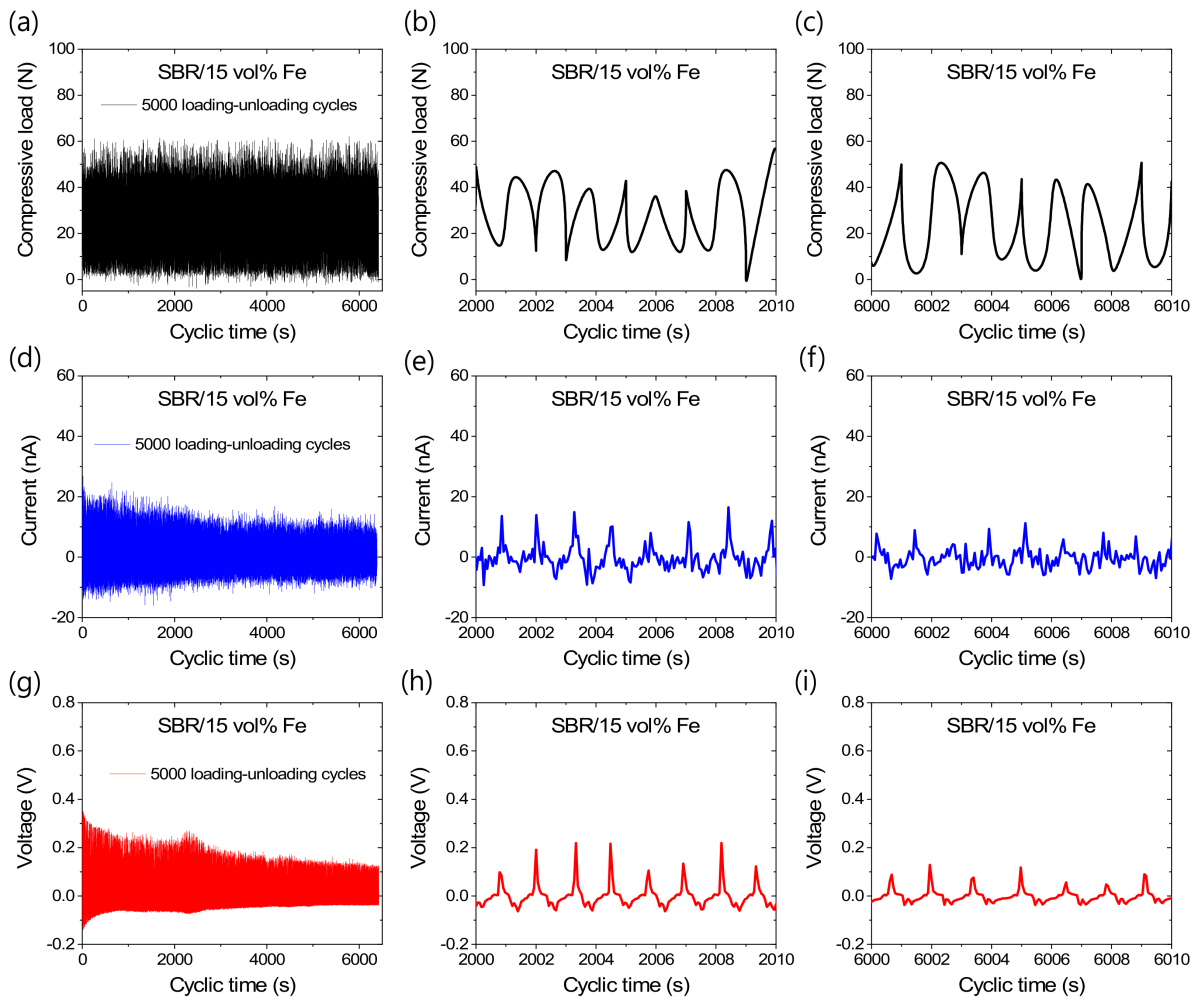
Mechanical and triboelectric fatigue properties of the SBR/15 vol% Fe rubber composite under 3% compressive loading–unloading cycles: (**a**) mechanical load degradation over 5000 continuous loading–unloading cycles, (**b**) mechanical load degradation during cycles from 2000 to 2010 s, (**c**) mechanical load degradation during cycles from 6000 to 6010 s, (**d**) triboelectric current degradation over 5000 continuous loading–unloading cycles, (**e**) triboelectric current degradation during cycles from 2000 to 2010 s, (**f**) triboelectric current degradation during cycles from 6000 to 6010 s, (**g**) triboelectric voltage degradation over 5000 continuous loading–unloading cycles, (**h**) triboelectric voltage degradation during cycles from 2000 to 2010 s, and (**i**) triboelectric voltage degradation during cycles from 6000 to 6010 s.

The fatigue behavior under 3% compressive strain over 5000 loading–unloading cycles is presented in Figure 10a–i. As shown in Figure 10a–c, no degradation in mechanical strength is observed during prolonged cyclic deformation, indicating excellent retention of mechanical properties.

Similarly, no significant degradation in the triboelectric current and voltage outputs is observed up to 2000 s of cyclic operation, as shown in Figure 10d,g, confirming the stable and reliable performance of the composites under repeated mechanical loading. However, prolonged continuous cycling results in a gradual reduction in both current and voltage outputs, as demonstrated in Figure 10d–i.

During repeated cyclic loading and unloading, viscoelastic energy dissipation can generate localized heat, which is known to adversely affect triboelectric charge generation [52]. DMA revealed that the loss modulus of the rubber composites decreases significantly with increasing temperature, indicating weakened filler–rubber interfacial interactions at elevated temperatures. Since interfacial interactions play a critical role in triboelectric charge generation, maintaining strong interfacial coupling is essential for sustained electrical output. Therefore, composites with lower heat accumulation or higher thermal conductivity are expected to preserve stronger interfacial interactions and maintain higher triboelectric performance.

In the present composites, the relatively high electrical and thermal conductivity of the metallic fillers is expected to promote rapid heat dissipation and thermal equilibration, thereby minimizing thermal accumulation and fatigue-induced degradation during continuous operation up to 2000 s. Beyond this cyclic duration, excessive heat generation at the filler–rubber interfaces may weaken interfacial interactions, leading to reduced charge generation efficiency. Therefore, intermittent cyclic deformation with appropriate recovery periods may be more favorable than uninterrupted continuous cycling for maintaining long-term triboelectric performance. Nevertheless, from a mechanical durability perspective, these rubber composites can withstand a large number of deformation cycles because the applied low compressive strain does not induce significant structural damage within the composite network.

Humidity is another important environmental factor affecting triboelectric performance. Figure S5a–f present the current and voltage outputs under 1% compressive cyclic deformation at different relative humidity conditions. The electrical outputs remain nearly unchanged between 30% and 50% relative humidity, whereas a slight reduction in current and voltage is observed at 80% relative humidity. SBR is inherently hydrophobic, and in the composite structure, the filler particles are encapsulated by rubber chains, resulting in minimal hydration of the filler–rubber interfaces. Therefore, moisture adsorption is expected to primarily occur at the electrode–composite interface, where excessive humidity can reduce triboelectric efficiency by facilitating charge dissipation. These results demonstrate the excellent moisture resistance of the developed rubber composite, suggesting its suitability for triboelectric energy harvesting applications under diverse environmental humidity conditions.

Since energy conversion is more efficient at lower compressive stress due to reduced heat dissipation, thin rectangular rubber sheets (0.01 cm thick, 5 × 6 cm^2^) of the SBR/15 vol% Fe composite were used instead of thick cylindrical samples to investigate current and voltage outputs in different TENG configurations employing a layer-by-layer electrode–sample structure.

**Figure 11:**
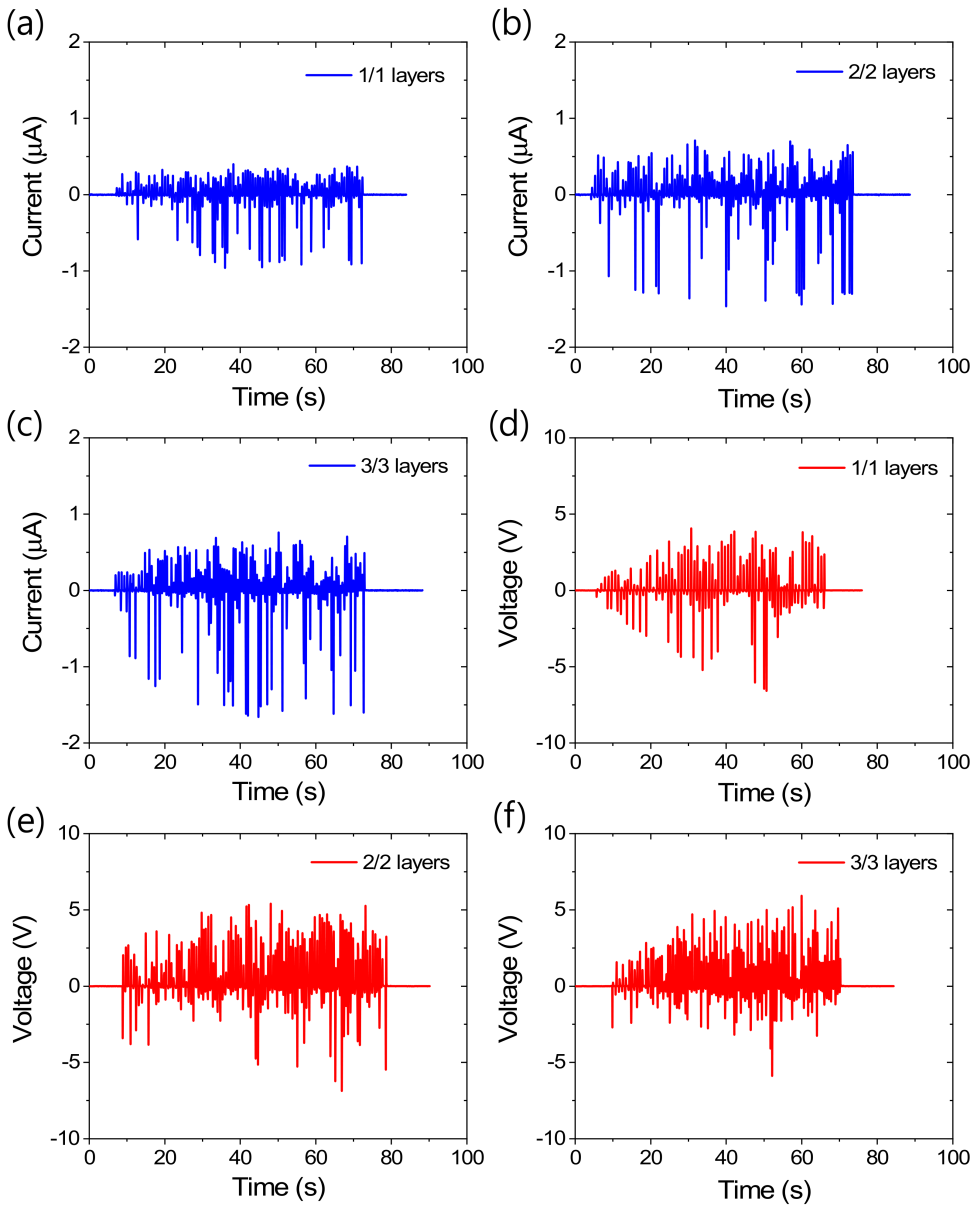
Triboelectric current and voltage outputs of SBR/15 vol% Fe composite sheets under hand patting in different layer-by-layer TENG structures: (**a**) current with 1/1 layers, (**b**) current with 2/2 layers, (**c**) current with 3/3 layers, (**d**) voltage with 1/1 layers, (**e**) voltage with 2/2 layers, and (**f**) voltage with 3/3 layers.

The current and voltage outputs generated by hand patting on the layer-by-layer TENGs are presented in Figure 11a–f. As shown in Figure 11a–c, the peak current intensity increases significantly for the 2/2-layer configuration compared to the 1/1-layer configuration and then becomes nearly constant for the 3/3-layer configuration. In contrast, the peak voltages shown in Figure 11d–f remain almost unchanged across the different layered TENG configurations.

These results indicate that both peak current and voltage outputs can reach the microampere and volt ranges, respectively, due to the increased surface area of the composite sample. Overall, these findings demonstrate that layer-by-layer TENG architectures can be effectively implemented to achieve higher energy generation with reduced mechanical energy loss during harvesting.

### 3.4. Mechanistic Aspects in Filler–Rubber Interactions and Charge Generation

To elucidate the physicochemical interactions between the fillers and the rubber matrix, XPS analyses were performed on the Al- and Fe-filled rubber composites. As shown in the major spectra presented in Figure S6a–f, the Zn 2p and S 2p peaks in the Fe-filled composite exhibit slight shifts toward lower binding energies compared with those in the Al-filled composite. This observation suggests that Fe particles possess stronger interactions with the curing components. Because Fe contains partially filled outer d-orbitals, it may participate in interactions with sulfur-based curing species during vulcanization. However, the enhanced reactivity of Fe with curing additives can consume active sulfur species, resulting in a reduced cross-link density, as observed in our previous investigation [37].

**Figure 12:**
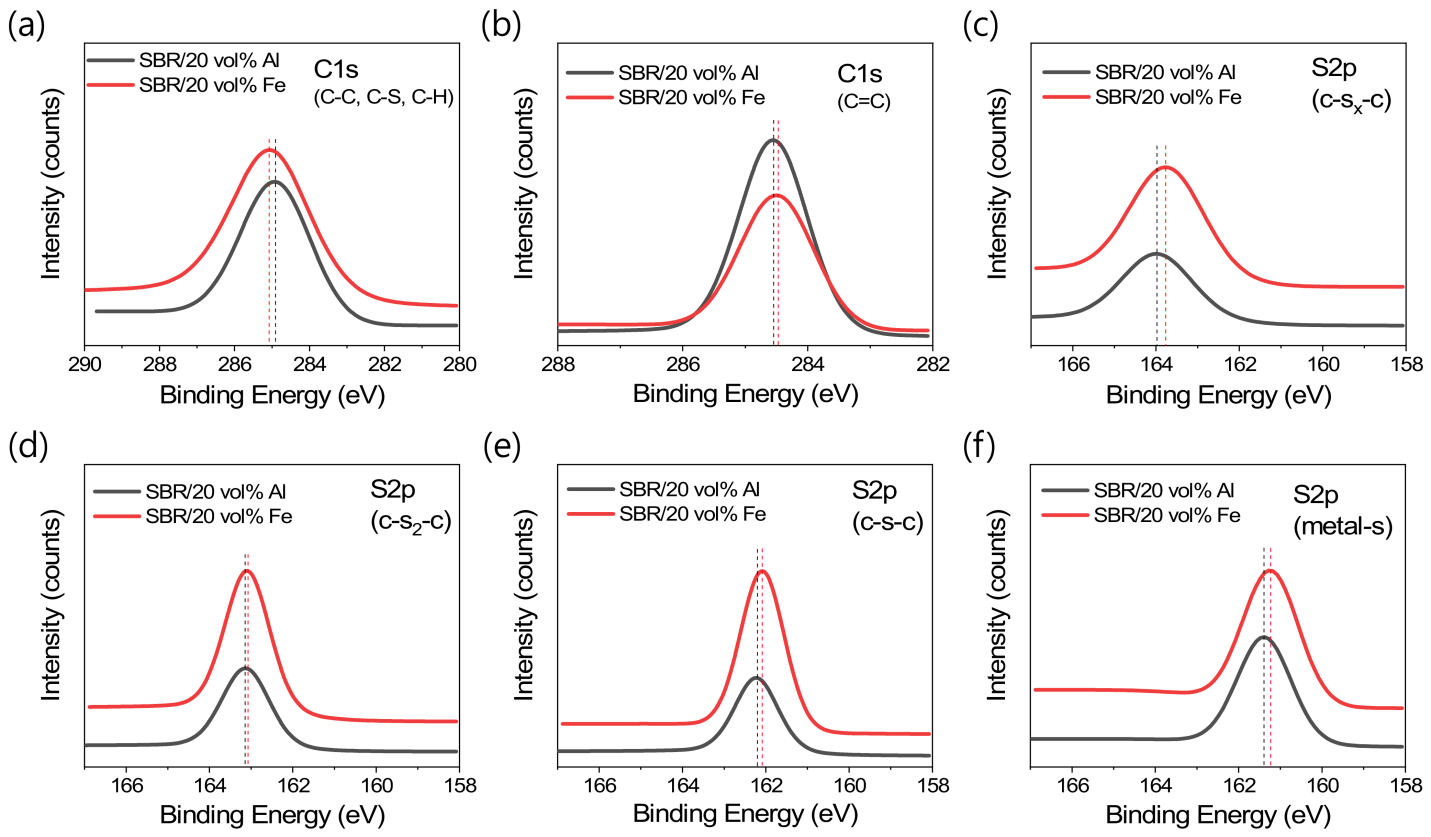
Deconvoluted XPS spectra of C 1s and S 2p with different chemical bonding environments (dotted lines indicate the peak position): (**a**) C 1s for C–C, C–S, and C–H bonds; (**b**) C 1s for C=C bonds; (**c**) S 2p for C–Sx–C bonds; (**d**) S 2p for C–S_2_–C bonds; (**e**) S 2p for C–S–C bonds; and (**f**) S 2p for metal–S bonds.

To further understand the interaction mechanism between Fe and the rubber matrix, the C 1s and S 2p spectra were deconvoluted and analyzed, as presented in Figure 12a–d. The C 1s spectrum of SBR can be primarily divided into two components corresponding to saturated carbon species and aromatic/unsaturated carbon species. As shown in Figure 12a, the C 1s peak associated with saturated carbon shifts toward higher binding energy in the Fe-filled composite, indicating a reduction in electron density around these carbon atoms. In contrast, the C 1s peak corresponding to aromatic and unsaturated carbon species shifts toward lower binding energy, suggesting an increase in electron density. These opposite binding energy shifts support the possible involvement of d–π orbital interactions between Fe atoms and the π-electron-rich phenyl groups of the SBR backbone. However, because the interacting atoms are spatially separated within the polymer–filler system, these interactions are expected to be relatively weak and primarily manifested as electrostatic interactions.

Similarly, Fe particles interact with sulfur-containing cross-linking structures, leading to increased electron density around sulfur atoms and a corresponding decrease in S 2p binding energy, as shown in Figure 12c–e. The different S 2p components were assigned according to their specific sulfur chemical environments within the vulcanized rubber network. For polysulfide cross-links, sulfur atoms exhibit characteristic S 2p peaks at 163.98 and 163.78 eV for Al- and Fe-filled composites, respectively. These values are close to the reported binding energy of elemental sulfur (S^0^, ~163.9 eV), confirming the presence of polysulfidic sulfur species [53]. The gradual decrease in S 2p binding energy with decreasing sulfur rank can be attributed to the enhanced electron-donating effect of adjacent carbon atoms.

Figure 12f presents the S 2p spectra associated with metal–sulfur bonding environments. During vulcanization, ZnO activators react with sulfur-containing accelerators to form thiocarbamate intermediates [54]. Since sulfur atoms in thiocarbamate structures possess a higher negative charge density, they exhibit the lowest binding energy among the sulfur species present in the vulcanized rubber network. Therefore, S 2p peaks located within the 162–161 eV range can be assigned to thiocarbamate sulfur species. Reported S 2p binding energies for ZnS and FeS are approximately 161.6 and 161.1 eV, respectively [53]. In the present study, the corresponding peaks appear at 161.4 and 161.2 eV for Al- and Fe-filled composites, respectively (Figure 12f).

The reduced cross-link density observed in Fe-filled composites suggests that Fe does not act solely as a conventional curing activator like ZnO but instead interacts strongly with sulfur species during vulcanization, consuming a fraction of the active sulfur and modifying the cross-linking process. The significant shift in the S 2p peak toward lower binding energy in the Fe-filled composite compared with the Al-filled composite provides strong evidence of enhanced physicochemical interactions between Fe fillers and the sulfur-crosslinked SBR network. These filler–rubber interactions contribute to the unique balance between reduced cross-link density and enhanced interfacial reinforcement observed in Fe-filled rubber composites.

To understand further the physicochemical interactions between the phenyl groups in rubber and metallic fillers, Raman spectroscopy was performed. Figure S7 presents the Raman spectra of unfilled SBR, as well as SBR/20 vol% Al and SBR/20 vol% Fe rubber composites.

SBR exhibits a characteristic Raman peak at 920.4 cm^−1^, which can be attributed to the breathing mode of the phenyl functional group [55,56]. Since SBR contains a relatively low amount of styrene units and the phenyl groups are distributed in a disordered manner along the rubber chains, this characteristic peak may appear with lower intensity and at a slightly lower frequency compared to polymers with higher styrene content [55,56].

As shown in Figure S7, the incorporation of metallic fillers induces a shift in this characteristic peak toward higher wavenumbers, indicating enhanced polarization effects arising from the interaction between the metallic fillers and the rubber matrix. The most significant peak shift is observed for the Fe-based rubber composite (~15 cm^−1^), compared to a relatively small shift (~3 cm^−1^) for the Al-based composite.

These results further suggest that Fe fillers may enhance the polarizability of the phenyl groups in SBR through possible d–π orbital interactions. However, due to the relatively large spatial separation between the interacting orbitals, these interactions are expected to be weak in nature.

**Figure 13:**
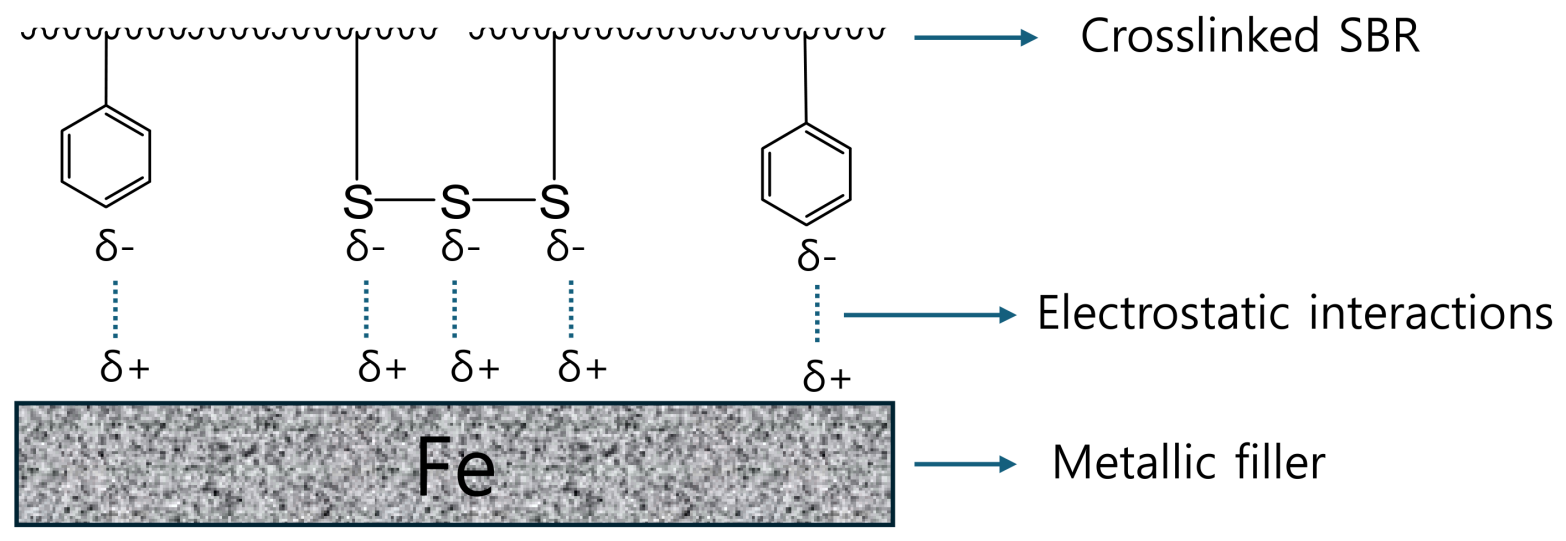
Schematic representation of the proposed electrostatic interactions between Fe particles and cross-linked SBR matrix.

Based on the rheological properties, XPS, and Raman spectroscopic analyses, Fe fillers exhibit stronger electrostatic interactions with the SBR matrix, resulting in enhanced reinforcement of the rubber composites. The proposed filler–rubber electrostatic interaction mechanism in the Fe-filled composites is illustrated in Figure 13. Since stronger interfacial electrostatic interactions facilitate triboelectric charge generation, Fe-filled composites demonstrate superior triboelectric energy-harvesting performance compared with Al-filled rubber composites.

**Figure 14:**
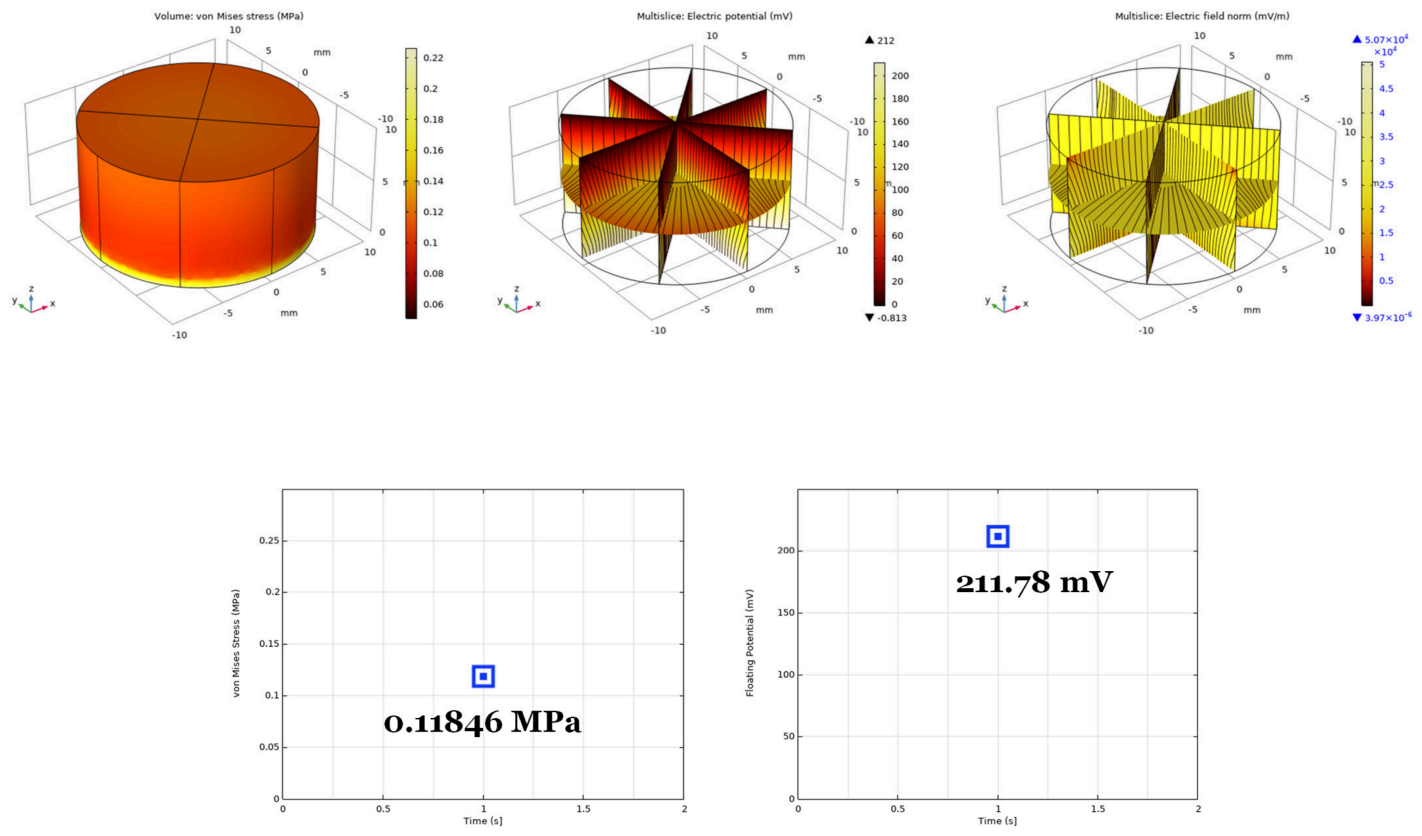
COMSOL simulation results of von Mises stress, electric potential, and electric field norm.

To elucidate the correlation between the mechanical properties and charge generation behavior, COMSOL (version 6.2.) simulations were performed for the SBR/15 vol% Fe composite under a compressive strain of 3%. The simulation results are presented in Figure 14. As observed, the von Mises stress is primarily concentrated at the rubber composite–electrode interface and gradually decreases toward the interior of the rubber specimen. Similarly, the electric potential exhibits a decreasing gradient with increasing depth within the rubber matrix. The simulated von Mises stress of 0.118 MPa and electric potential of 211.78 eV show good agreement with the experimentally obtained values.

Because rubber is a viscoelastic material, the applied mechanical stress can be transmitted and redistributed throughout the polymer matrix, inducing deformation at internal filler–rubber interfaces and contributing to the overall triboelectric charge generation. Therefore, these results indicate that both the electrical characteristics and viscoelastic properties of rubber composites play critical roles in determining triboelectric energy-harvesting performance.

## 4. Conclusions

In this study, aluminum (Al)- and iron (Fe)-filled styrene–butadiene rubber (SBR) composites were systematically developed and evaluated for triboelectric energy harvesting applications. The results demonstrate that the incorporation of metallic fillers significantly influences the mechanical, electrical, and triboelectric properties of the composites, with Fe-filled systems consistently outperforming their Al-filled counterparts.

Mechanical characterization revealed that Fe-filled composites exhibit superior reinforcement, higher tensile strength, and greater fracture toughness, indicating stronger filler–rubber interactions. Electrical analyses showed that Fe-filled composites approach a percolation threshold at approximately 15 vol% filler loading, resulting in substantially enhanced electrical conductivity, low impedance modulus and higher capacitance. In contrast, Al-filled composites displayed negligible conductivity owing to weaker interfacial interactions and high impedance in the composite.

The triboelectric performance was found to be strongly dependent on the synergistic effects of electrical conductivity, capacitance, and interfacial physicochemical interactions. Among all compositions, the composite containing 15 vol% Fe exhibited the highest energy-harvesting performance, achieving a peak current density of 127.05 µA m^−2^, a transferred charge of 5.01 nC, and a peak power density of 48.22 µW m^−2^ under 1% compressive strain. These improvements are attributed to enhanced electrostatic interactions at the filler–rubber interface, arising from interactions between Fe particles and the benzene rings as well as sulfur cross-links within the SBR matrix. These interfacial interactions are further supported by XPS and Raman spectroscopic analyses.

Furthermore, the developed composite demonstrated excellent mechanical and triboelectric durability under repeated cyclic loading and had good humidity resistance. The successful implementation of thin layer-by-layer TENG architectures further highlights the feasibility of achieving efficient and scalable energy harvesting while minimizing mechanical energy dissipation.

Overall, this work establishes the critical importance of filler–polymer physicochemical interactions in governing triboelectric performance and identifies Fe-filled SBR composites as promising materials for high-performance, durable, and cost-effective triboelectric nanogenerators and self-powered sensing devices. Future studies will focus on hybrid filler systems incorporating nanoscale reinforcing additives to further enhance the mechanical robustness, electrical characteristics, and environmental stability of the composites for operation under demanding service conditions.

## Figures and Tables

**Table 1:** Mix compositions of rubber composites.

Formulation	Amount of Masterbatch Rubber in g	Amount of Aluminum in g (vol%)	Amount of Iron in g (vol%)
SBR-unfilled	25	-	-
SBR/10 vol% Al	25	8.0 (10) *	-
SBR/15 vol% Al	25	12.7 (15)	-
SBR/20 vol% Al	25	18.0 (20)	-
SBR/10 vol% Fe	25	-	23.3 (10)
SBR/15 vol% Fe	25	-	37.0 (15)
SBR/20 vol% Fe	25	-	52.3 (20)

* The vol% of fillers was calculated considering the density of masterbatch rubber (0.94 g/cm^3^) and the theoretical densities of aluminum (2.7 g/cm^3^) and iron (7.87 g/cm^3^).

**Table 2:** Summarization of mechanical and charge generation characteristics under 1% compressive cyclic strain.

Formulation	Mechanical Load (N)	Total Charge (nC)	Charge to Load Ratio (nC/N)
SBR-unfilled	7.286	1.242	0.171
SBR/10 vol% Al	5.842	1.288	0.221
SBR/15 vol% Al	6.101	1.488	0.244
SBR/20 vol% Al	6.742	1.646	0.245
SBR/10 vol% Fe	5.325	4.193	0.788
SBR/15 vol% Fe	5.405	5.009	0.927
SBR/20 vol% Fe	6.931	4.496	0.649

## Data Availability

The original contributions presented in this study are included in the article/Supplementary Material. Further inquiries can be directed to the corresponding author.

## Supplementary Materials

The following supporting information can be downloaded at: https://www.mdpi.com/article/10.3390/polym18151801/s1, Figure S1: Schematic illustration of the triboelectric nanogenerator (TENG) setup used for mechanical loading experiments.; Figure S2: Compressive load–deformation curves of (a,b) Al-based rubber composites and (c,d) Fe-based rubber composites.; Figure S3: Electrical properties of rubber composites; (a) capacitance of Al-based composites, (b) capacitance of Fe-based composites, (c) resistance of Al-based composites, and (d) resistance of Fe-based composites.; Figure S4: Triboelectric voltage outputs of different rubber composites under loading–unloading cycles at increasing compressive strains: (a) SBR-unfilled, (b) SBR/10 vol% Al, (c) SBR/15 vol% Al, (d) SBR/20 vol% Al, (e) SBR/10 vol% Fe, (f) SBR/15 vol% Fe, and (g) SBR/20 vol% Fe.; Figure S5: Current and voltage outputs of SBR/15 vol% Fe at different humidities; (a,b) 30%, (c,d) 50%, and (e,f) 80% humidity.; Figure S6: XPS of different major elements in rubber composites; (a) Zn 2p, (b) O 1s, (c) C 1s, (d) S 2p, (e) Al 2p, and (f) Fe 2p.; Figure S7: Raman spectra of unfilled and filled rubber composites.
