# Supplementary material for: Metallic (Al and Fe) Powder-Reinforced Styrene–Butadiene Rubber Composites for Triboelectric Energy Harvesting
Source: Polymers (Basel). 2026 Jul 23;18(15):1801. doi: 10.3390/polym18151801 (PMC13467622; doi:10.3390/polym18151801)
Supplement: Supplementary file 1 [file polymers-18-01801-s001.zip › polymers-4399463-supplementary.pdf]

Supporting Information

Metallic (Al and Fe) Powder-Reinforced Styrene–Butadiene Rubber Composites for Triboelectric Energy Harvesting

Md Najib Alam, Vishnu Shankar Dhandapani, Sang-Shin Park*

School of Mechanical Engineering, Yeungnam University, 280 Daehak-ro,
Gyeongsan 38541, Republic of Korea

*Correspondence: S.-S. Park (email: pss@ynu.ac.kr)

1. **Experimental**

$$\text{Peak current density} = \frac{\Delta \text{I}}{Contact\ area\ of\ sample\ to\ electrode} \ldots\ldots\ldots\ldots\ldots\ldots \text{(S1)}$$

$$\text{Charge} = \int_0^t \text{I(t)dt} \quad \ldots\ldots\ldots\ldots\ldots\ldots\ldots \text{(S2)}$$

$$\text{Peak power density} = \frac{\Delta \text{V}.\Delta \text{I}}{Contact\ area\ of\ sample\ to\ electrode} \ldots\ldots\ldots\ldots\ldots\ldots\ldots \text{(S3)}$$

where ΔI is the sum of the negative and positive output peak currents in a single cyclic loading and unloading, I(t) is the current at time t, and ΔV is the sum of the negative and positive output peak voltages. For determining efficiencies, the average output from 10 cycles were considered.

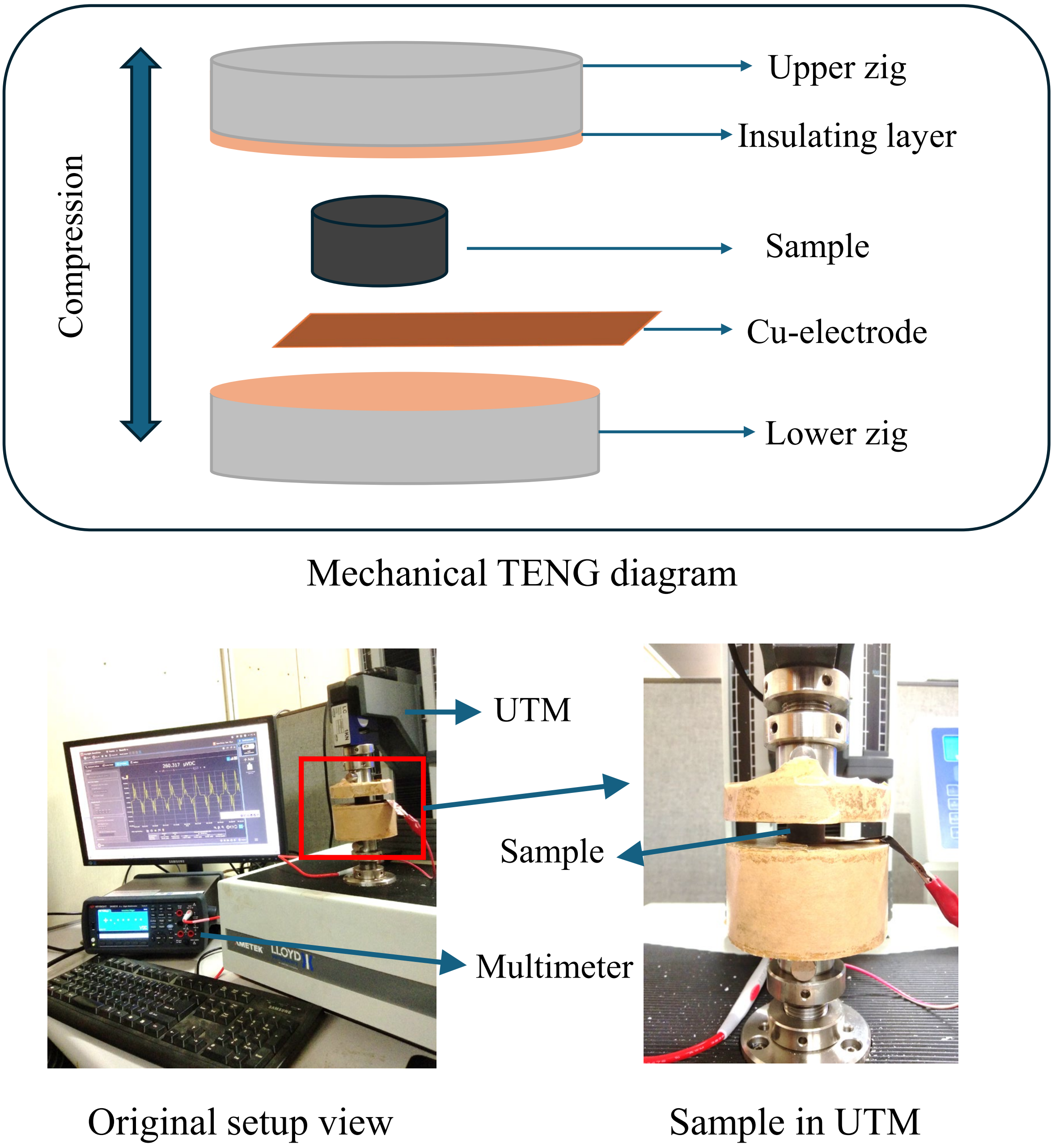

Figure S1: Schematic illustration of the triboelectric nanogenerator (TENG) setup used for mechanical loading experiments.

2. Results

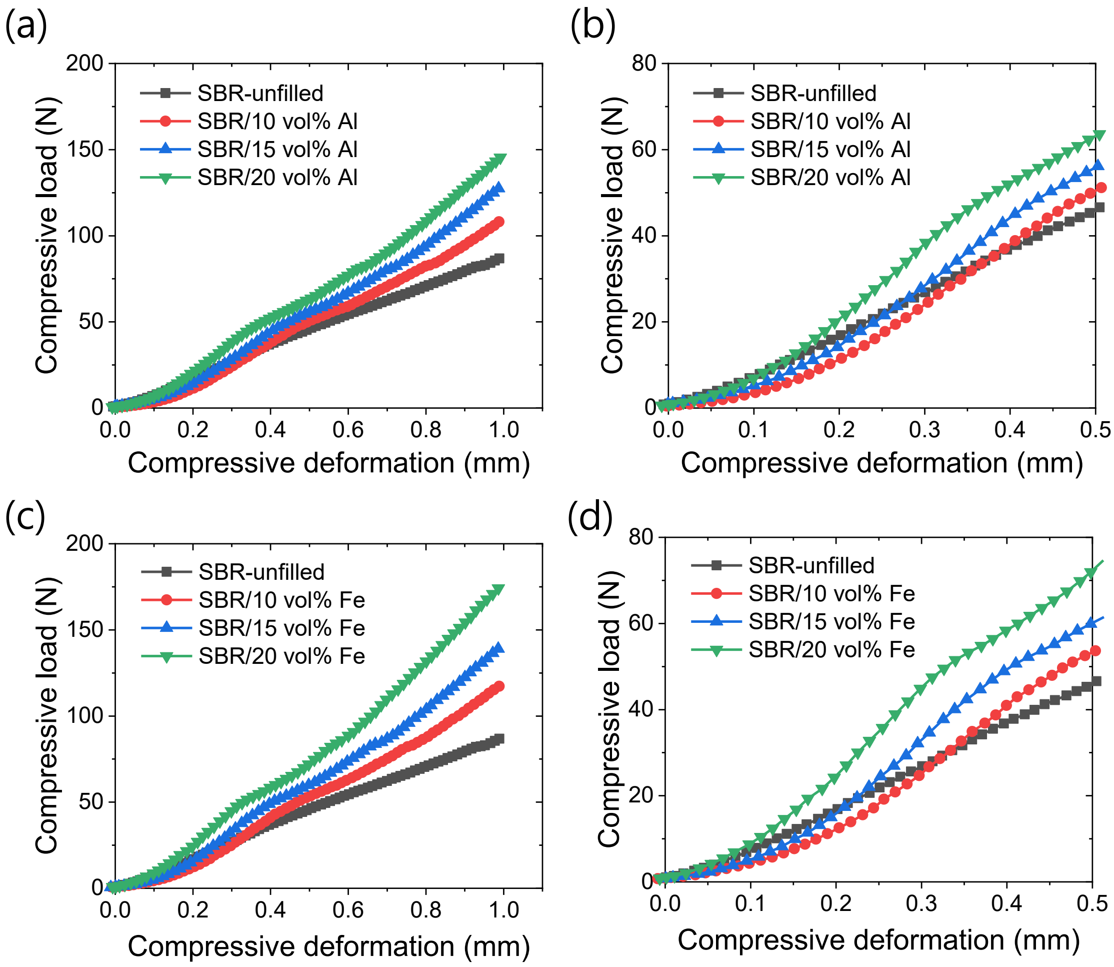

Figure S2: Compressive load–deformation curves of (a,b) Al-based rubber composites and (c,d) Fe-based rubber composites.

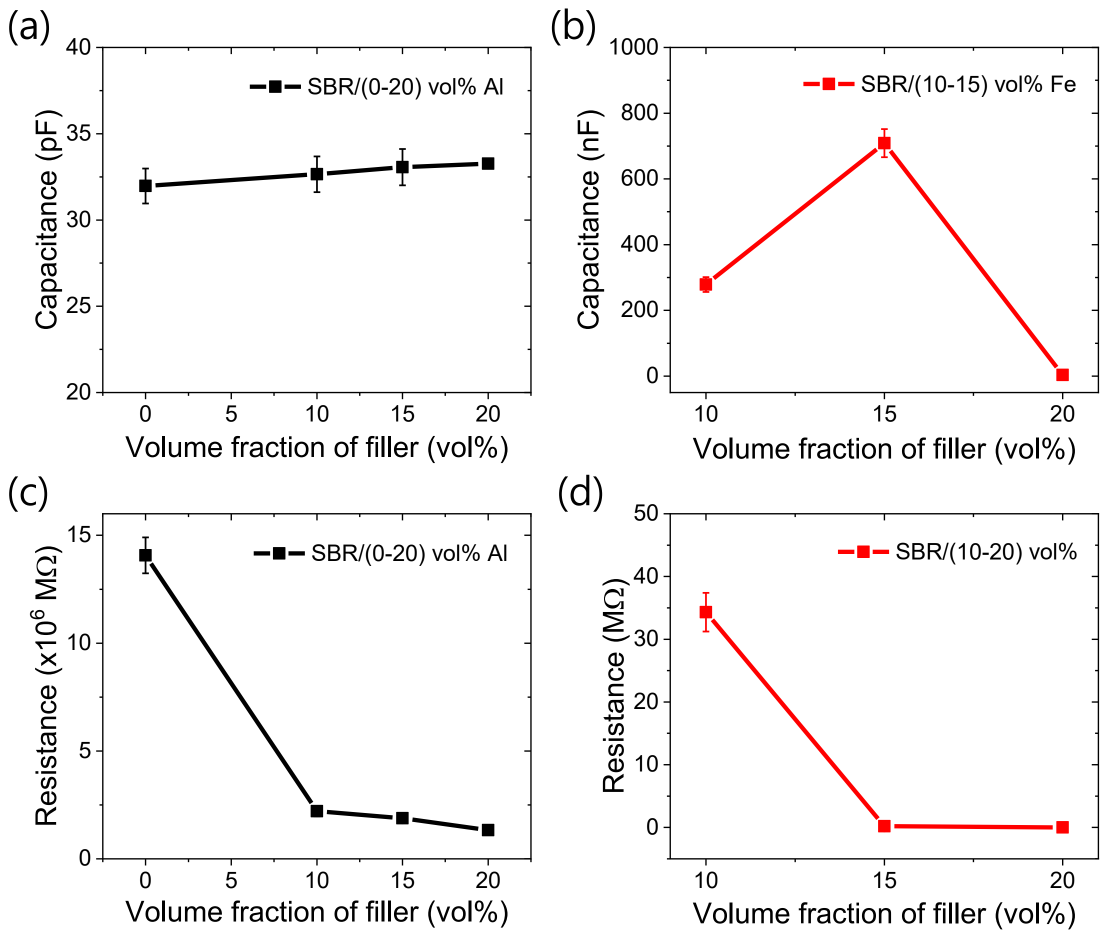

Figure S3: Electrical properties of rubber composites; (a) capacitance of Al-based composites, (b) capacitance of Fe-based composites, (c) resistance of Al-based composites, and (d) resistance of Fe-based composites.

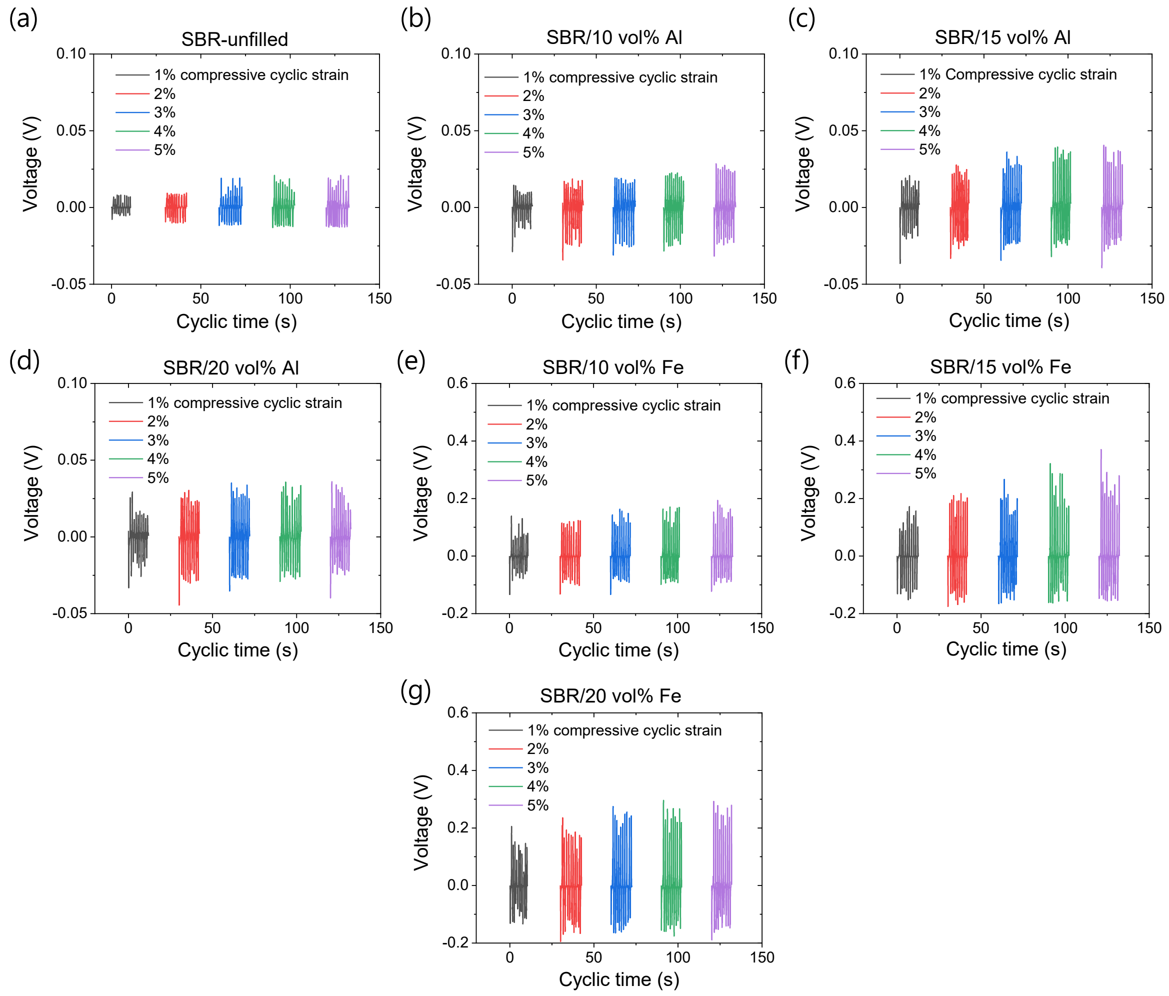

Figure S4: Triboelectric voltage outputs of different rubber composites under loading–unloading cycles at increasing compressive strains: (a) SBR-unfilled, (b) SBR/10 vol% Al, (c) SBR/15 vol% Al, (d) SBR/20 vol% Al, (e) SBR/10 vol% Fe, (f) SBR/15 vol% Fe, and (g) SBR/20 vol% Fe.

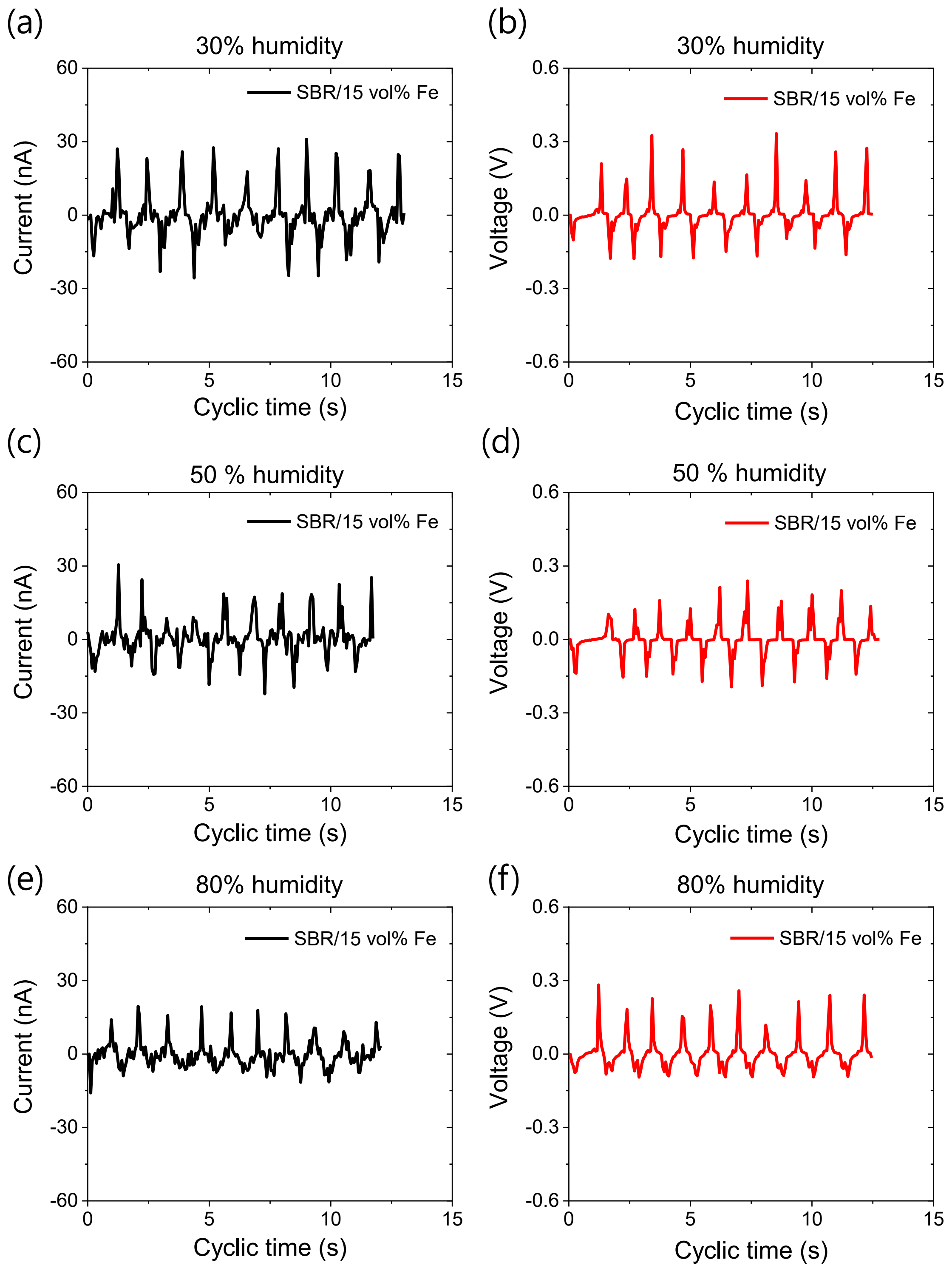

Figure S5: Current and voltage outputs of SBR/15 vol% Fe at different humidities; (a,b) 30%, (c,d) 50%, and (e,f) 80% humidity.

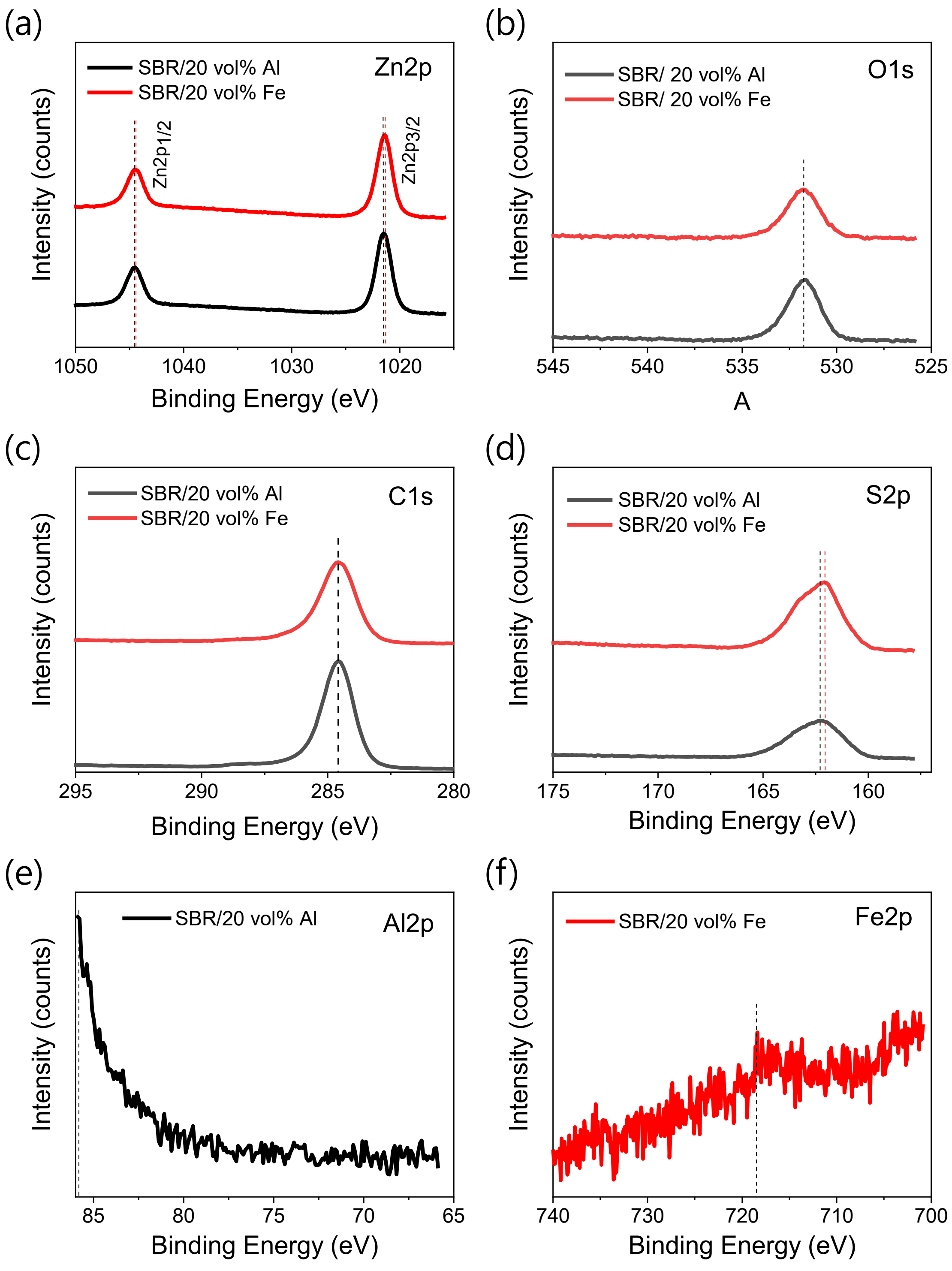

Figure S6: XPS of different major elements in rubber composites; (a) Zn 2p, (b) O 1s, (c) C 1s, (d) S 2p, (e) Al 2p, and (f) Fe 2p.

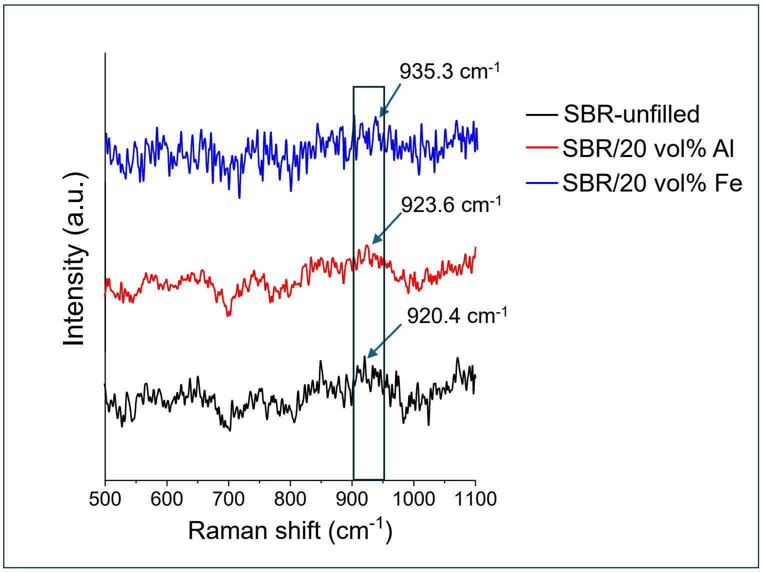

Figure S7: Raman spectra of unfilled and filled rubber composites.
